# Dams and Introduced Species Drive Patterns of Environmental Adaptation in an Iconic but Imperiled Coldwater Fish (Brook Trout, *Salvelinus fontinalis*)

**DOI:** 10.1111/eva.70239

**Published:** 2026-05-03

**Authors:** Nadya Mamoozadeh, Arthur Cooper, Henry Quinlan, Anna Varian, Dana Infante, Mariah Meek

**Affiliations:** ^1^ Department of Applied Ecology North Carolina State University Raleigh North Carolina USA; ^2^ Department of Integrative Biology Michigan State University East Lansing Michigan USA; ^3^ Department of Fisheries and Wildlife Michigan State University East Lansing Michigan USA; ^4^ (Retired) U.S. Fish and Wildlife Service Ashland Fish and Wildlife Conservation Office Ashland Wisconsin USA; ^5^ Stantec Woodbury Minnesota USA; ^6^ The Wilderness Society Bozeman Montana USA

**Keywords:** brook trout, climate change, environmental adaptation, habitat fragmentation, landscape genomics

## Abstract

Freshwater biodiversity is being disproportionately negatively impacted by anthropogenic stressors including climate change, partly due to limited opportunities to seek more favorable conditions compared to marine and terrestrial species. Management plans that maintain locally adapted genotypes, and integrate active management interventions where needed, are vital for curbing further biodiversity loss but require information on environmental adaptation. Here, we explore environmental adaptation in native populations of a cold‐adapted freshwater species (brook trout, *Salvelinus fontinalis* ) with a long history of negative impacts from human‐induced stressors and now increasingly affected by climate change. We developed a new restriction site‐associated DNA capture panel to genotype 3297 SNPs in over 2200 brook trout from 55 waterways across the Lake Superior basin. We used partial redundancy analyses to identify over 300 SNPs potentially involved in adaptation. Our study is among the first to implicate both movement barriers and introduced species in shaping environmental adaptation, along with climate. System‐specific variables for introduced Pacific salmon and stream fragmentation caused by movement barriers exhibited some of the strongest associations with genetic variation, highlighting the role that dams and introduced species—features characteristic of the Great Lakes and much of North America—may play in shaping adaptation in native fishes. We also identified temperature and stream flow as strongly associated with putatively adaptive genetic variation, as reported in other studies. Importantly, these insights were possible given locally derived environmental information, which we analyzed alongside widely accessible broad‐scale climate data. Finally, by calculating adaptive index and genomic offset, we identified areas where brook trout appear heterogeneously adapted across the landscape and where locally adapted genotypes may be prone to disruption from climate change. Our findings offer novel insights into environmental adaptation in brook trout and foundational knowledge for developing management plans that conserve the adaptive capacity of populations already experiencing changing conditions.

## 1 Introduction

Growing impacts of climate change and myriad anthropogenic stressors on wild populations present an urgent need to understand how species are adapted to their environment. Knowledge of environmental adaptation, including involved regions of the genome and primary selective forces, is necessary for conserving locally adapted genotypes and predicting how species may be affected by changing conditions (Franks and Hoffmann 2012; Rausher and Delph 2015). This information is vital to develop conservation plans that support the resiliency and long‐term persistence of species, for example by identifying at‐risk populations which may be candidates for active intervention (Flanagan et al. 2018; Gaitán‐Espitia and Hobday 2021; Meek et al. 2023). Genomic datasets offer a useful mechanism for exploring environmental adaptation (Bernatchez et al. 2023; Harrisson et al. 2014; Hoffmann et al. 2015; Stapley et al. 2010; Theissinger et al. 2023), and a rapidly growing number of genomic studies have greatly expanded our understanding of how species are adapted to their local environment (Bay et al. 2018; Hung et al. 2023; Mahony et al. 2020; Moreira and Smith 2023; Pritchard et al. 2018; Tigano et al. 2024), providing practical information to guide conservation planning.

Although datasets describing genome‐wide variation are becoming increasingly common, species‐specific data on environmental variables potentially relevant to genetic adaptation are logistically challenging to collect and are often unavailable at fine geographic scales, especially for freshwater species that inhabit many fragmented habitats. So far, many studies of genetic adaptation in species of conservation or management concern leverage broad‐scale climate datasets from widely accessed public repositories (e.g., WorldClim [https://www.worldclim.org/], AdaptWest Project (2015) [https://adaptwest.databasin.org/]), which play an indispensable role in enabling such investigations. These datasets often comprise climate variables likely to be important drivers of adaptation across many species and are particularly valuable given the availability of data on historical and future conditions. However, for many populations there may also be system‐specific factors that play an important role in environmental adaptation, and failure to identify these factors will undermine the effectiveness of efforts to conserve adaptive variation. For example, ecological factors such as the presence of introduced species affect native populations (Bernery et al. 2022; Emery‐Butcher et al. 2020; Hansen et al. 2019; Hitt et al. 2017), but these factors remain largely unexplored in the context of genetic adaptation. Studies that incorporate locally derived information are important for facilitating comprehensive insights into adaptation in local systems.

Species inhabiting freshwater systems are particularly vulnerable to anthropogenic stressors given their limited ability to track changing conditions by moving to new environments, among other factors (Dudgeon 2019; Dudgeon et al. 2006; Woodward et al. 2010). Extinction rates for riverine fishes are estimated to be more than 100 times higher than natural extinction rates in some regions, potentially due to fragmentation and introduced species (Dias et al. 2017). Dispersal opportunities in freshwater habitats are increasingly restricted by fragmentation; for example, 79% of stream length in the conterminous United States is disconnected from its downstream outlet by a dam (Cooper et al. 2017). In the Great Lakes, culverts are 38 times more frequent than dams (Januchowski‐Hartley et al. 2013), reflecting another potentially important factor contributing to the fragmentation of freshwater habitats. Fragmentation has been shown to negatively affect freshwater biodiversity, including native populations of trout (Cooper et al. 2017; Kovach et al. 2022; Roberts et al. 2013; Torterotot et al. 2014), many species of which are considered sensitive indicators of habitat quality. Trout populations are expected to be further negatively impacted by climate change and other anthropogenic stressors (Bell et al. 2021; Isaak et al. 2012; Kovach et al. 2019), especially warming stream temperatures (Roberts et al. 2013; Mitro et al. 2019), which collectively pose a multifaceted threat to native trout populations. Identifying and mitigating negative impacts from these threats is especially critical for preserving trout species, which often support economically valuable commercial and recreational fisheries, are culturally important to Indigenous communities, and provide important ecological functions to native aquatic ecosystems (Power 1980).

One species with a long history of human‐mediated impacts that is now increasingly affected by climate change is the brook trout ( *Salvelinus fontinalis* ), a coldwater trout native to northeastern North America and the Laurentian Great Lakes. In the eastern United States, brook trout have been extirpated from nearly one‐third of native subwatersheds, primarily due to habitat loss (Hudy et al. 2008). In the Great Lakes basin, more than 50% of native brook trout habitat may be lost in future decades due to changing climatic conditions (Stewart et al. 2016). Recent analysis of brook trout across the native range (Meek et al. 2025) revealed adaptive genetic signatures strongly associated with temperature that varied by geographic region, with many areas exhibiting some degree of genomic offset (Capblancq and Forester 2021; Fitzpatrick and Keller 2015) to future climate conditions. Some of the genetic variants associated with these patterns were also implicated in a thermal stress experiment performed on a subset of populations (Meek et al. 2025). Additional studies have identified variables for temperature, elevation, and the presence of other salmonids as influencing environmental adaptation in native populations of brook trout (Ferchaud et al. 2020; Jeon et al. 2025). Findings from these and similar studies have spurred increasing efforts toward developing management plans that better account for factors affecting the conservation status of brook trout and the effectiveness of management interventions. However, additional information on the ecological and evolutionary effects of climate change and other anthropogenic stressors on native populations is needed to guide conservation actions in local waterways.

Among the diverse ecological and environmental variables potentially shaping adaptation in brook trout are movement barriers, both natural and human‐made, which can significantly impact habitat structure and genetic diversity in freshwater systems (Zarri et al. 2022). In particular, dams impose localized and landscape‐scale disturbances on stream habitats, including through altered flow and temperature regimes, sedimentation rates, and habitat availability (Cooper et al. 2017; Zarri et al. 2022). Potential evolutionary consequences of population fragmentation due to decreased connectivity between above‐ and below‐barrier reaches include reduced genetic diversity, increased inbreeding, and diminished adaptive potential accompanied by elevated extinction risk (Frankham et al. 2017). In native brook trout, reduced levels of genetic diversity have been reported for above‐barrier populations, especially when habitat is limited and population size is small (Michaelides et al. 2025; Torterotot et al. 2014; Whiteley et al. 2013). Habitat fragmentation can also alter the selective regime, particularly for small populations, resulting in greater levels of adaptive divergence among populations (Fraser et al. 2014). Understanding how movement barriers affect adaptive genetic diversity is vital for mitigating negative impacts of dams and other structures on the adaptive potential of brook trout and other stream species.

Here, we surveyed genome‐wide variation in native populations of brook trout across a large spatial extent to accomplish four primary objectives: (1) identify regions of the genome that potentially form a genetic basis to adaptation, (2) identify broad‐scale climate and localized environmental variables that may act as locally important drivers of adaptation, (3) infer the spatial distribution of putative adaptive genetic variation across the landscape, and (4) predict the level of genomic offset to future climate conditions. To accomplish these objectives, we compared inferences based on environmental variables that incorporate locally derived information relative to modeled climate data collected at a broad spatial scale. Additionally, we compared inferences among different types of barriers (e.g., natural vs. human‐made) that potentially affect upstream and downstream movements of brook trout. Our findings not only shed light on environmental adaptation in native populations of brook trout but also offer insights into factors that play an important role in adaptation to freshwater habitats yet are seldom considered in studies aimed at inferring adaptive relationships.

## 2 Methods

### 2.1 Landscape Genomic Datasets

#### 2.1.1 Field Sampling and Genomic Data

**FIGURE 1:**
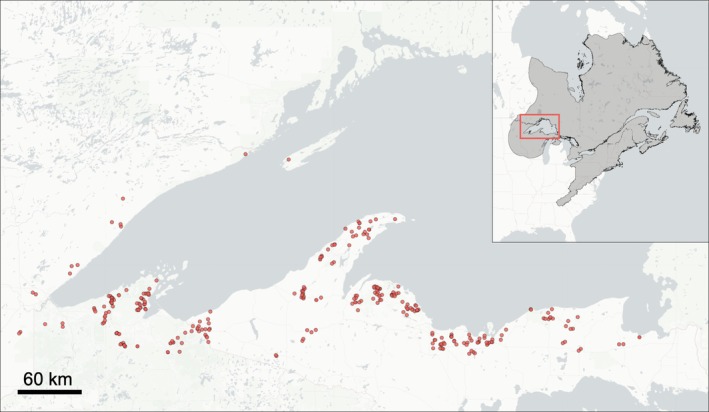
Map depicting the sampling locations for the brook trout retained in the dataset used for genotype–environment association analyses. Points reflect sampling sites within the Lake Superior basin listed in Table S1. Inset shows expanded view of surrounding geographic region within the brook trout native range (shaded area).

We analyzed a dataset comprising genotypes at genome‐wide single nucleotide polymorphisms (SNPs) surveyed in brook trout across the Lake Superior basin (Table S1 and Figure 1). The Lake Superior basin supports brook trout exhibiting different migration propensities, including resident brook trout which spend their life in stream habitats and endemic migratory brook trout characterized by spending a portion of their life in Lake Superior (Huckins et al. 2008). These migratory brook trout, known as coasters, were historically abundant across the basin but today occur in comparatively few locations. For practicality, fisheries managers in the region distinguish brook trout captured in lake habitats as coasters (Newman et al. 2003), although some coasters are sympatric with nonmigratory brook trout in stream habitats during the spawning season. Unless otherwise noted, nearly all of the brook trout evaluated in this study were sampled from stream habitats, including at sites substantially upstream of connections to Lake Superior, and were thus presumed to primarily consist of nonmigratory brook trout.

The genomic data evaluated here were produced using a restriction site‐associated DNA (RAD) capture panel (Ali et al. 2016) newly developed in this study. Briefly, this panel was designed using 248 brook trout from 63 waterways across the full extent of the Lake Superior basin, including four areas within the lake, and 42 domestically raised brook trout from nine hatchery strains stocked in the region (Tables S1, S2 and Figure S1; see Data S1: for detailed methods on panel development). Some of the streams sampled here are known or suspected to be seasonally inhabited by migratory brook trout (e.g., Pilgrim River, Adams 2020; Zorn, Pangle, et al. 2024; Zorn, Rudh, and Gerig 2024), Salmon Trout River (Scribner et al. 2012), Nipigon River and other rivers tributary to Nipigon Bay (D'Amelio et al. 2008; Elias et al. 2018). The SNPs targeted in our RAD capture panel were discovered by conducting RAD sequencing following Ali et al. (2016), where sequence data were aligned to the *Salvelinus* sp. reference genome (Christensen et al. 2018; but see Christensen et al. 2021) prior to SNP calling. We used this reference because the panel design and genotype–environment analyses described here were completed before a reference genome for brook trout became available (Lecomte et al. 2025). Our RAD capture panel targets 33,309 SNPs that reflect a wide range of genetic diversity in brook trout native to the Lake Superior basin, making it a useful tool that can be leveraged in future studies to efficiently genotype brook trout from this region.

We used the RAD capture panel created here to conduct high‐throughput genotyping of brook trout across the US portion of the Lake Superior basin. Through this effort, we produced a quality filtered dataset containing over 2200 brook trout (Tables S1, S2 and Figure 1; see Data S1 for additional details) for exploring population‐level genetic relationships. The brook trout in this dataset were primarily sampled during the months of June through September in the years 2008, 2010–2014, and 2016–2017 using backpack electrofishing in streams, with most samples collected in 2011. Brook trout were sampled from 58 streams and rivers tributary to Lake Superior and at four locations within the lake. We conducted sampling at 1–17 sites (mean = 5 sites) in each river or stream, where sampling efforts targeted all life stages. We conducted additional filtering of the quality filtered SNP dataset to yield a dataset useful for the genotype‐environment analyses described below (see Data S1). We excluded hatchery reared brook trout (*n* = 406) and wild‐caught brook trout from sites where either local environmental data (*n* = 110 individuals) or broad‐scale climate data (*n* = 132 individuals) were unavailable. We also excluded loci that exhibited a minor allele frequency < 1% to reduce the level of importance attributed to rare alleles in ordination analyses. Finally, missing genotypes were imputed using the k‐nearest neighbor genotype imputation method (Money et al. 2015) implemented in Tassel 5 (Bradbury et al. 2007). We used this dataset to explore basic population genetic relationships among brook trout sampled from different waterways, including by calculating pairwise genetic differentiation (Fst; Nei 1987), observed and expected heterozygosities (Ho, He), and inbreeding coefficient (Fis) using hierfstat (Goudet 2005), before conducting a suite of genotype–environment analyses as described below.

#### 2.1.2 Locally Derived Environmental Data

We compiled a suite of variables from the US portion of the Lake Superior basin that reflect localized environmental factors potentially affecting adaptation in brook trout, including stream habitat fragmentation, human landscape alteration, hydroclimatic conditions, population demographics, and stocking of brook trout and non‐native species of Pacific salmon. These variables included 18 metrics measuring stream network fragmentation due to the presence of dams and naturally occurring waterfalls, which were calculated at the level of stream reach or patches within stream catchments defined using barrier locations (Cooper et al. 2017; Table S3). Briefly, these include barrier densities, distances to barriers, and total stream network availability based on the summed distances of connected stream reaches (i.e., patch distances). These metrics were derived using stream reaches from the National Hydrography Dataset (NHD) Plus v2 geospatial dataset (McKay et al. 2012) and characterize the influence of dams and waterfalls on fragmentation and flow at both local and landscape scales. We separately derived these metrics for large dams (generally > 2 m in height) listed in the National Anthropogenic Barrier Dataset (Ostroff et al. 2013), small dams (provided by state regulatory agencies), and waterfalls (Wieferich 2016), as well as for all three barrier types together. We thus evaluated four sets of stream fragmentation metrics, meaning that we assessed multicollinearity among variables and conducted variance partitioning and redundancy analyses (RDA; described below) separately for each barrier type (e.g., analyses inclusive of locally derived variables were performed a total of four times).

To characterize human landscape alteration, we evaluated variables describing land cover and use, human population density, and road crossings within catchments containing sample locations. We also assessed variables describing hydroclimatic conditions, including precipitation, air temperature, and stream baseflow, as well as catchment area and flowline slope. We assembled 49 variables from the 2011 National Land Cover Database (Homer et al. 2015) that were derived for local and network catchments (Table S3). Local catchments are defined as the land area draining directly to a stream reach, while network catchments include the entire upstream land area contributing to a stream reach (Wang, Infante, Esselman, et al. 2011).

In addition to variables describing habitat and landscape conditions, we also evaluated variables reflecting population demographic and ecological relationships potentially affecting environmental adaptation in brook trout. These included two categorical variables that describe the demographic population status of brook trout at the catchment and 12‐digit hydrologic unit code (HUC) subwatershed levels (U.S. Fish and Wildlife Service (USFWS) 2016; Table S3). For these variables, population status was either directly inferred using information on the proportion of brook trout relative to non‐native salmonids and number of life history stages present, or indirectly inferred via model predictions (U.S. Fish and Wildlife Service (USFWS) 2016). This consisted of self‐sustaining vs. nonself‐sustaining population classes at the catchment level and intact vs. nonintact population classes for HUC‐12s. These composite variables—aggregated at two spatial scales—are potentially relevant to environmental adaptation because demographically informed conservation status may be reflective of genetic diversity, including adaptive diversity, which may be influenced by demographic factors, such as census size and age structure, within populations. We also included two binary variables that describe the presence of brook trout relative to naturalized non‐native salmonids historically and currently stocked in the Great Lakes (U.S. Fish and Wildlife Service (USFWS) 2016; Table S3). These variables are potentially relevant to environmental adaptation because introduced species may affect genetic diversity in brook trout and the spatiotemporal distribution of this diversity through differential resource competition, fitness, and survival, among other possible avenues. For example, experimental studies show that brook trout can be outcompeted by coho salmon for food (Thornton et al. 2017), and experience habitat displacement due to the presence of coho salmon, brown trout, or rainbow trout (Fausch 1988; Fausch and White 1981, 1986; Hitt et al. 2017). Data for all of the variables described here were collected at each of the US sites where brook trout were sampled in this study.

Finally, we derived variables from hatchery stocking records for domestic brook trout stocked into the Lake Superior basin during the period 1998–2017. After compiling all available records within the US extent of the Lake Superior basin, we removed records for stocking events occurring in isolated ponds to minimize the influence of stocking unlikely to have affected the stream‐sampled brook trout analyzed here. To identify stocking locations within isolated ponds, we visually compared stocking locations with water bodies and stream reaches from the NHDPlus v2 dataset. After removing stocking records from isolated ponds, we used remaining records to derive two variables reflecting stocking effort near sampling sites (Table S3). We did this by summarizing stocking events that occurred near each site (within an 800 m radius), so that the spatial scale of these variables corresponded with sampling sites.

#### 2.1.3 Climate Data

In addition to localized environmental variables, we separately evaluated broad‐scale climate variables based on modeled climatic conditions and widely accessed via a public database. We assessed 27 bioclimatic variables developed by AdaptWest using the ClimateNA database (Table S4; Wang et al. 2016; Available from: https://adaptwest.databasin.org/pages/adaptwest‐climatena‐cmip5/). These variables describe seasonal and annual patterns in air temperature and precipitation. Variables for contemporary climate were based on standardized climate normals for the years 1961–1990. Given an average generation time of 3–4 years, these baseline climate data were collected at least five generations before sampling occurred for most of the brook trout evaluated in this study (2011). Future climate was based on average projections from 15 CMIP5 models (Knutti et al. 2013). We evaluated projected climate based on Representative Concentration Pathways (RCP) emissions scenarios RCP4.5 and RCP8.5 (intermediate and extreme scenarios, respectively) at the years 2050 and 2080. We assessed future climate based on RCP projections (rather than other projections, such as Shared Socioeconomic Pathways [SSPs]) to evaluate effects of changes in the physical climate under different emissions trajectories.

### 2.2 Climate and Environmental Variable Reduction and Scaling

In preparation for subsequent analyses, we assessed variable multicollinearity and rescaled variables for both the broad‐scale climate and localized environmental variables. We controlled for multicollinearity within each dataset by retaining only one variable from sets of two or more variables that exhibited Pearson correlation coefficients > |0.7|. A threshold of > |0.7| is widely reported in the literature for identifying excessively correlated variables prior to genotype‐environment association analyses (Capblancq et al. 2020; Layton et al. 2021; Micheletti et al. 2018). For the four datasets containing locally derived variables (one dataset per barrier type), we retained the same variables across datasets as much as possible. For some sample locations, localized environmental data were missing for a subset of variables. In these cases, we imputed missing values within each dataset using the knnImputation function of the DMwR2 package (Torgo 2016) in R, which uses a weighted mean calculated from *k* nearest neighbors. Finally, we centered and scaled values for continuous variables within each dataset.

### 2.3 Partitioning the Influence of Climate and Environment on Genetic Variation

To identify categories of variables with greater explanatory power for genetic variation in brook trout, we used partial RDA (pRDA; Capblancq and Forester 2021) to conduct a series of variance partitioning analyses. We used variance partitioning to estimate the proportion of genetic variance attributed to each of seven variable categories (Table S3) while controlling for the influence of remaining variables (Capblancq and Forester 2021; Legendre and Legendre 2012). After developing a full model with all variables, we conducted pRDAs where variables from one category were used as predictors in the model and remaining variables were retained as covariates. This determined the proportion of genetic variance independently explained by each category (Capblancq and Forester 2021). To control for the effects of sampling geography and neutral population structure, we also included variables for sampling site longitude (due to the east–west orientation of sample sites) and the first two axes from a principal component analysis based on neutral loci. We identified loci likely to be selectively neutral using pcadapt (Luu et al. 2017) where we used a false discovery rate of 40% to distinguish outlier loci and leave behind a set of conservatively identified neutral loci. Pcadapt identifies outliers with respect to population structure and is ideally suited for hierarchically structured populations given its use of statistically robust Mahalanobis distances to distinguish outliers (Luu et al. 2017). These factors are important for brook trout, which frequently exhibit large degrees of population structure that is also often hierarchical (this study; Mamoozadeh et al. 2023).

Bias can be introduced when categories comprising differing numbers of variables are used for variance partitioning (Borcard et al. 1992). We assessed the potential impact of this bias by conducting a permutation analysis where we randomly selected four predictor variables from the four categories that contained more than four variables. We then used the subsampled variables within each category to calculate explanatory power. We performed 100 permutation iterations then compared permutation results to results from categories that were not subsampled. We conducted all variations of pRDA using the vegan package (Oksanen et al. 2022) in R.

### 2.4 Identifying Loci That Potentially Form a Genetic Basis to Adaptation

We next used pRDA to conduct genotype–environment association analyses aimed at identifying loci that potentially form a genetic basis to adaptation as well as associated environmental drivers (Capblancq and Forester 2021; Forester et al. 2018). Genotype‐environment association methods assume that environmental clines drive corresponding allele frequency clines. However, this relationship may not be monotonic due to the complexity of multivariate environments (Lotterhos 2023), and identified environmental variables may only be correlated with the true causal agent of selection (Capblancq and Forester 2021). Regardless, these methods are a widely used and effective tool for assessing adaptation. In the pRDA models we tested, genotypes were modeled as the multivariate response to a suite of predictor variables. We also included conditioning variables for neutral population structure to control for the effect of neutral structure on association analyses. After estimating the pRDA model, we used the first two RDA axes to calculate the Mahalanobis distance of each locus (Capblancq and Forester 2021; Capblancq et al. 2018). We then corrected Mahalanobis distances for the inflation factor (François et al. 2016), transformed these distances to *p* values (Luu et al. 2017), and identified loci with a *p* value below a Bonferroni‐corrected threshold of 1% as outliers that potentially form a genetic basis to adaptation. We conducted pRDA separately for the broad‐scale climate variables and the localized environmental variables.

### 2.5 GO Term Enrichment of Outlier Loci

We evaluated whether any known biological processes were over‐represented in regions of the genome containing the putatively adaptive SNPs identified using pRDA. BEDTools (Quinlan 2014) was used to search regions extending 10 kbp upstream and downstream of outlier and non‐outlier SNPs for gene ontology (GO) annotations in the *Salvelinus* sp. reference genome. We then used the topGO R package (Alexa and Rahnenführer 2024) to test for functional enrichment of GO terms in regions containing outlier SNPs. A node size of five and the weight01 algorithm were employed in topGO analyses. We applied a correction (Benjamini and Hochberg 1995) to resulting *p* values then used a significance threshold of 1% to identify over‐represented GO terms. This analysis was repeated a second time using annotations from zebrafish ( *Danio rerio* ). We used OmicsBox with default settings to conduct a functional analysis that included a blastp search against *D. rerio* in the NCBI reference database, followed by extracting the top blast hit and exporting GO annotations prior to analyzing GO terms in topGO (Altschul et al. 1990; Götz et al. 2008).

### 2.6 Adaptation to Contemporary and Future Conditions

We used the relationship between outlier SNPs and predictor variables to generate adaptive landscapes based on contemporary and future projected conditions. We conducted an adaptively enriched pRDA (aeRDA; Capblancq and Forester 2021) limited to the outlier loci identified via pRDA, then used this aeRDA model to infer a contemporary adaptive landscape by calculating adaptive index (Capblancq and Forester 2021; Steane et al. 2014) for locations across our study system. Adaptive index reflects the amount of adaptive similarity or difference across a landscape and is calculated independently for each aeRDA axis and pixel within the landscape. For aeRDA based on the climate predictors, this adaptive index corresponded with pixels spanning the Lake Superior basin. In comparison, for aeRDA based on the localized environmental variables, we were only able to calculate adaptive index for pixels containing sampling sites because complete environmental data were unavailable for locations beyond these sites. Next, we calculated genomic offset (Bay et al. 2018; Capblancq and Forester 2021; Fitzpatrick and Keller 2015; Rellstab et al. 2016) by comparing the aeRDA relationship based on contemporary conditions with an aeRDA performed using future projected climate. Genomic offset reflects the degree to which gene–environment relationships may be disrupted due to environmental change (Fitzpatrick and Keller 2015) and is calculated as the Euclidean distance between the adaptive index estimated from contemporary vs. future environmental data (Capblancq and Forester 2021).

Our goal for the genomic offset analysis was to evaluate the amount of genetic change that may be needed for brook trout to adapt to new environments given climate change. This analysis is valuable because it identifies areas on the landscape where locally adapted genetic diversity is more or less likely to be disrupted by a changing climate. Genomic offset provides fisheries managers with information on populations expected to be more robust against change, which may function as strongholds, and populations expected to be more heavily impacted by change, which may be at risk of extirpation without targeted intervention. We estimated genomic offset by calculating the difference between the optimal adaptive index under current vs. future conditions (Capblancq and Forester 2021). We calculated genomic offset only for the dataset composed of broad‐scale climate predictors because future projections were unavailable for localized environmental variables.

## 3 Results

### 3.1 RAD Capture Dataset

Our final quality filtered SNP dataset comprised 3297 SNPs genotyped at 2251 individuals in the dataset inclusive of localized environmental variables and 2229 individuals in the dataset based on broad‐scale climate variables (Table S2). On average, 70 SNPs were retained per chromosome (range = 8–156 SNPs) in the *Salvelinus* sp. reference genome assembly, with an average of 1 SNP retained (range = 1–9 SNPs) per scaffold. The average Ho and He per waterway was 0.124 (range = 0.061–0.714; Figure S2) and 0.133 (range = 0.063–0.187), respectively, and average Fis was 0.047 (range = −0.072–0.205). Pairwise Fst averaged 0.083 (range = 0.006–0.425; Figure S2), reflecting negligible to large degrees of genetic differentiation between the waterways sampled in this study.

### 3.2 Landscape Genomic Datasets

We produced a suite of datasets useful for exploring environmental adaptation in brook trout native to the Lake Superior basin. For the localized environmental data, we produced four datasets that corresponded with each of the barrier types we evaluated (e.g., small dams, large dams, waterfalls, and all barrier types combined). After controlling for multicollinearity (Tables S5–S14), we retained a total of 48 environmental predictors within each dataset (Table S3), except for the dataset with combined barrier types, where we retained 52 predictors. These environmental predictors corresponded with 2251 brook trout from 55 waterways containing 280 sites across the Lake Superior basin that were genotyped at 3297 SNPs (Table S1, Figure 1 and see Data S1 for additional details). For the climate variables, we produced a dataset that comprised seven predictors (Tables S4, S14) after controlling for multicollinearity (Tables S4, S13, S14). These climate predictors corresponded with 2229 brook trout from 54 waterways containing 278 sites that were genotyped at 3297 SNPs (Table S1, Figure 1 and see Data S1 for additional details). The sites analyzed in the datasets inclusive of locally derived environmental information were identical to those analyzed in the dataset based on broad‐scale climate, except for two sites that were missing in the latter dataset. We used each of the datasets described here to model genotypes as the multivariate response to predictor variables in variance partitioning (pRDA and aeRDA).

### 3.3 Partitioning the Influence of Climate and Environment on Genetic Variation

We used variance partitioning to identify categories of predictor variables that explain comparatively large proportions of genetic variance and that may shape adaptation in brook trout native to the Lake Superior basin. Results from variance partitioning conducted using the predictor variables inclusive of locally derived information were similar across barrier datasets (Tables 1, S15–S17); therefore, we only describe results from the dataset based on combined barrier types (Table 1). Our full model indicated that predictors for localized environment, neutral structure, and sampling geography collectively explained 12% of genetic variance (*p* < 0.001). The four categories containing variables for stream fragmentation, hydroclimatic conditions, and land cover each had a significant effect (*p* < 0.001), despite each explaining about 1% of total genetic variance (8.38%–10.54% of explainable variance). These categories also contained the largest numbers of predictors, and permutation tests based on four randomly selected variables within each category resulted in an average explanatory power (adjusted *R* ^2^) of 0.23%–0.49% (average of 3.30%–5.52% of explainable variance; Figure S3), which was still higher than remaining predictor categories. Neutral structure exhibited a larger effect on genetic variance than any other predictor category (2.14% and 18.17% of total and explainable variance, respectively; Table 1). Nearly half of explainable variance (47.31%) was attributed to an interaction among predictor categories.

**TABLE 1:** Results from variance partitioning conducted using locally derived environmental variables and combined barriers.

Category color‐code	Model	*R* ^2^	Adjusted *R* ^2^	Variance (Total inertia)	Model *p* value (*p* (> F))	Percent explainable variance	Percent total variance
	Full model	0.1179	0.0958	61.30	0.001	100.00	11.79
	Fragmentation only	0.0124	0.0082	6.46	0.001	10.54	1.24
	Hydroclimatic only	0.0099	0.0072	5.14	0.001	8.38	0.99
	Land cover (local) only	0.0115	0.0064	5.96	0.001	9.72	1.15
	Land cover (network) only	0.0120	0.0077	6.23	0.001	10.16	1.20
	Population density only	0.0017	0.0009	0.89	0.001	1.45	0.17
	Road crossings only	0.0017	0.0009	0.90	0.001	1.47	0.17
	Population status only	0.0020	0.0012	1.04	0.001	1.70	0.20
	Hatchery stocking only	0.0037	0.0022	1.94	0.001	3.16	0.37
	Neutral structure only	0.0214	0.0211	11.14	0.001	18.17	2.14
	Geography only	0.0012	0.0008	0.62	0.001	1.01	0.12
	Confounded			29.00		47.31	5.58
	Total unexplained			458.42			88.21
	Total inertia			519.72			100.00

Variance partitioning conducted using the climate dataset revealed lower explanatory power than the dataset inclusive of locally derived information. Our full model included variables for climate, neutral structure, and sampling geography (Table 2). This model was significant (*p* < 0.001) and explained 7% of genetic variance, approximately 5% less than the model inclusive of local information. The climate predictors had a significant effect (*p* < 0.001) and explained 1.68% of genetic variance (24.18% of explainable variance). Neutral structure again had a greater effect on genetic variance than any other category (3.55% and 51.16% of total and explainable variance, respectively). Over 21% of explainable variance was confounded across predictor categories.

**TABLE 2:** Results from variance partitioning conducted using climate variables.

Model	*R* ^2^	Adjusted *R* ^2^	Variance (total inertia)	Model *p* (*p* (> *F*))	Percent explainable variance	Percent total variance
Full model	0.0694	0.0652	36.06	0.0001	100.00	6.94
Climate only	0.0168	0.0139	8.72	0.0001	24.18	1.68
Neutral population structure only	0.0355	0.0348	18.45	0.0001	51.16	3.55
Geography only	0.0024	0.0020	1.27	0.0001	3.52	0.24
Confounded climate/structure/geography			7.62		21.13	1.47
Total unexplained			483.37			93.06
Total inertia			519.43			100.00

### 3.4 Identification of Outlier Loci and GO Term Enrichment

**FIGURE 2:**
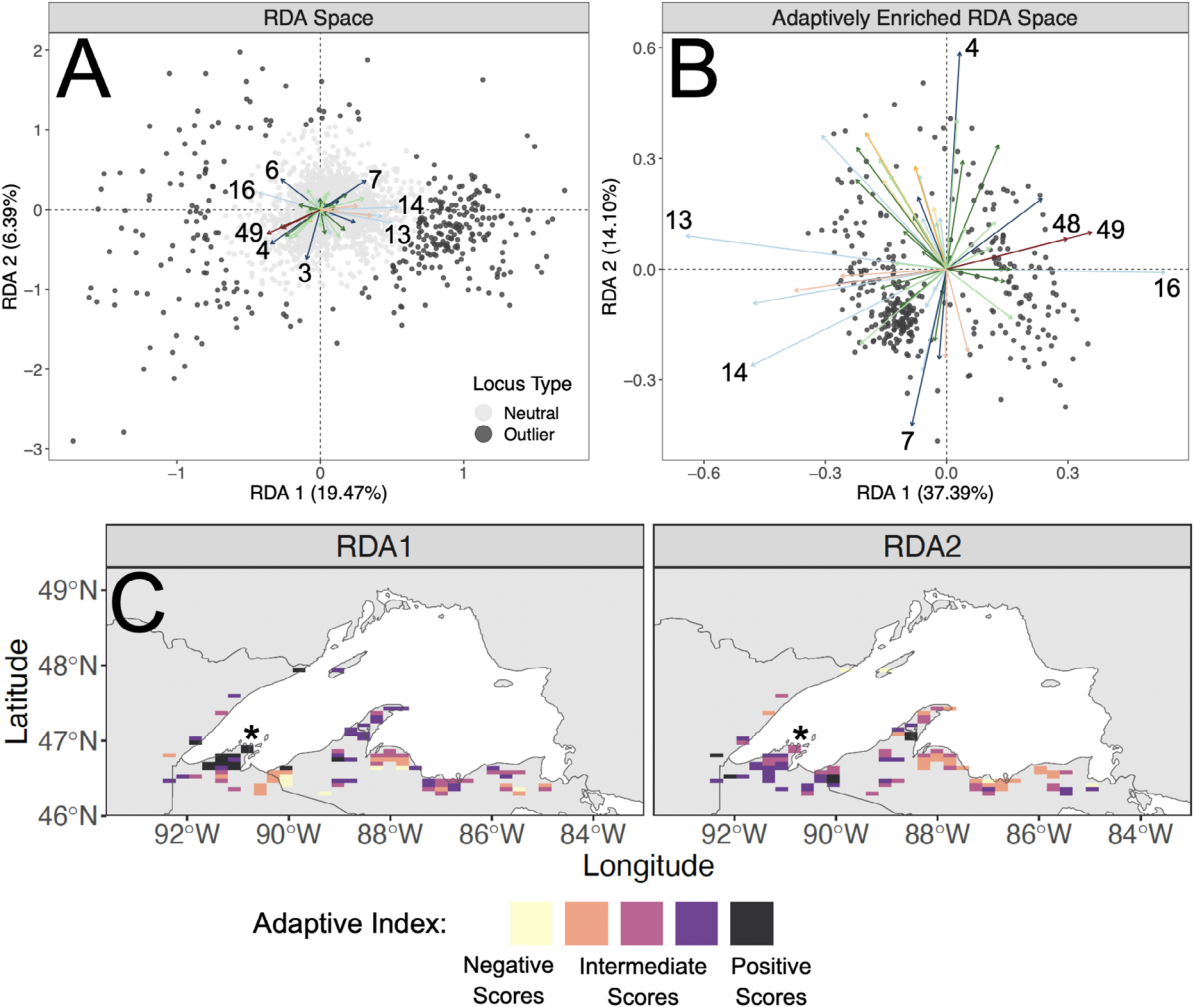
Results from RDA conducted using the dataset inclusive of locally derived environmental variables for combined barrier types. Vectors in Panels A and B are color‐coded as in Table 1, and numeric labels match the field numbers listed in Table S2. Panel A: Results from pRDA. Panel B: Results from aeRDA. Panel C: Contemporary landscape based on adaptive index calculated from aeRDA. The location of the Bayfield Peninsula is indicated with an asterisk.

**FIGURE 3:**
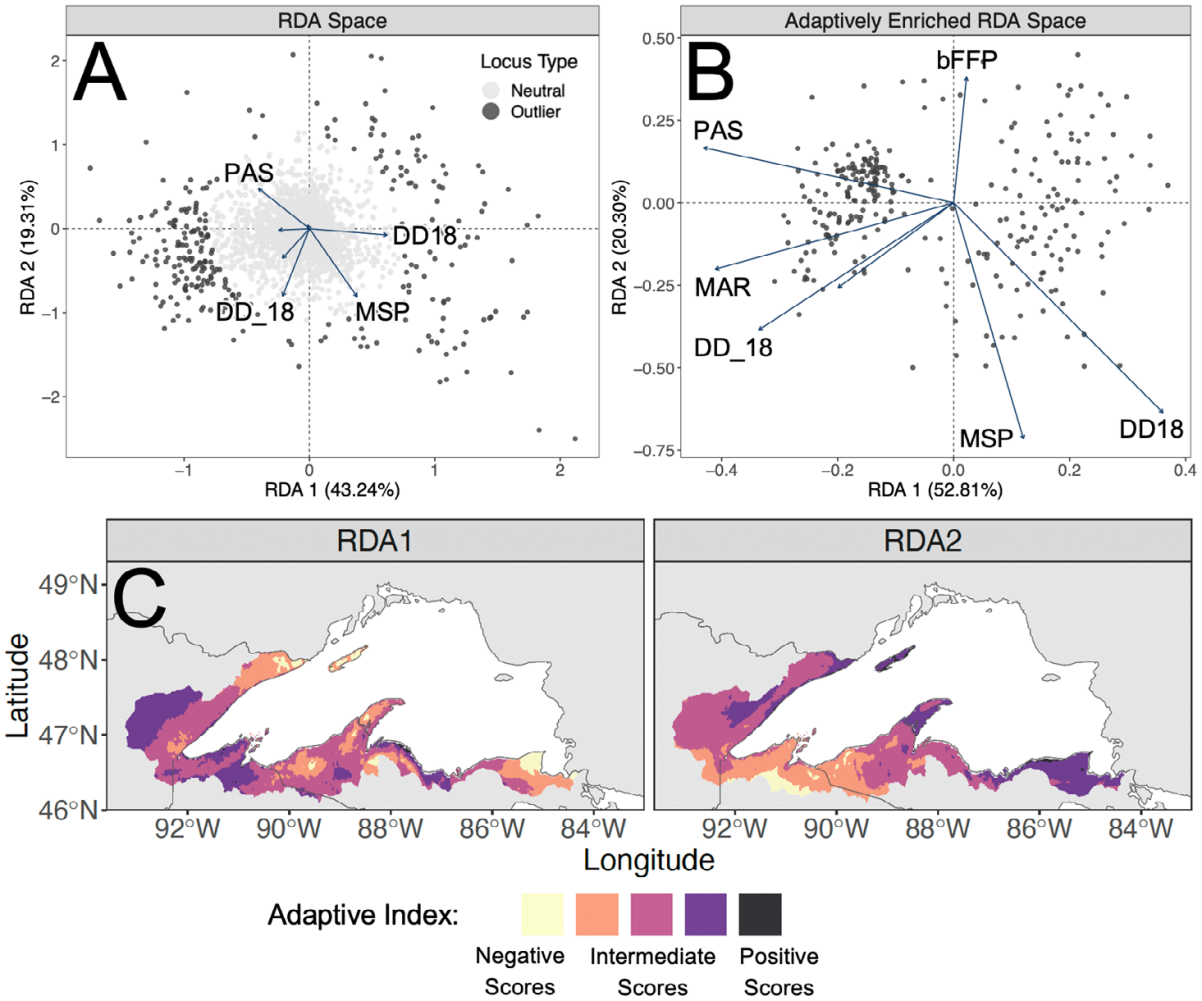
Results from RDA conducted using the dataset based on climate variables. Panel A: Results from pRDA. Panel B: Results from aeRDA. Panel C: Contemporary landscape based on adaptive index calculated from aeRDA.

We used results from pRDA to identify outlier SNPs that potentially form a genetic basis to adaptation in the brook trout analyzed here. We evaluated these relationships based on pRDA axes one and two. For the datasets based on localized environmental variables, these axes explained 18.94%–20.24% (axis one) and 6.39%–6.83% (axis two) of genetic variation across the four barrier datasets (Figures 2A, S4–S6). We identified 337–352 outliers from these four datasets; 312 of which were identified as outliers across all four barrier types (Figure S7). Additionally, 239 of these SNPs were also identified as outliers in results based on the climate dataset (Figure S7). In this dataset, axes one and two explained 43.23% and 19.31% of variation, respectively (Figure 3A). The outliers we identified through pRDA were distributed throughout the *Salvelinus* sp. reference genome (Figure S8). Additional outlier loci would likely be detected without a SNP panel, as used here, and if utilizing the recently available brook trout reference genome (Lecomte et al. 2025).

We used outliers identified from the dataset based on combined barrier types to identify GO terms in flanking regions and test for enrichment. We found a single GO term (GO:0042552) from the *Salvelinus* sp. reference genome that was identified as over‐represented based on unadjusted *p* values (*p* < 0.01; Table S18) and was related to myelination. Using the *D. rerio* genome, five additional GO terms were identified as over‐represented based on unadjusted *p*‐values (*p* < 0.01; Table S18). Gene functions for these GO terms related to immune response (B cell differentiation), development (skeletal system development, blood vessel morphogenesis), and intracellular signaling (kinase activity, MAPK cascade). For all six terms identified across both genomes, four or more significant genes were observed, representing a 1.5 to more than six‐fold increase over expected numbers of significant genes. However, none of the GO terms identified here remained significant after adjusting for multiple comparisons (*p* > 0.05).

### 3.5 Adaptation to Contemporary and Future Conditions

We identified variables that exhibited strong associations with outlier SNPs and potentially represent important aspects of the climate or local environment that drive adaptation. In results from aeRDA performed with the localized environmental predictors (Figures 2B, S9–S11), axis one explained 35.77%–39.63% of putatively adaptive genetic variation across the four barrier datasets and was most strongly driven by variables for demographic population status (variables 48 [catch_class] and 49 [huc12_class]; Table S3) and the presence of barriers (variables 10 [DownMainDens], 13 [TotalMainCnt], 14 [TotalMainDens], 16 [TotalMainOpen]; Table S3). A variable describing the presence of non‐native salmonids (variable 51 [non_native]; Table S3) exhibited the strongest associations with axis one in results from two of the barrier datasets (large dams and small dams; Figures S9, S10). In comparison, variables for summer air temperature (variable 4 [L_j_temp]; Table S3) and stream base flow (variable 7 [N_bfi]; Table S3) were the strongest drivers of axis two, which explained 12.29%–14.10% of genetic variation among outlier loci. In aeRDA results based on the climate dataset (Figure 3B), variables for temperature (MAR, DD18, DD_18; Table S4) and precipitation (PAS; Table S4) were the strongest drivers of axis one, which explained 52.81% of variation. Axis two explained 20.30% of variation and was also primarily driven by variables for precipitation (MSP; Table S4) and temperature (bFFP; Table S4).

Contemporary landscapes based on adaptive index differentiated adaptively distinct regions of the Lake Superior basin. These findings indicate that brook trout are heterogeneously adapted across the Lake Superior basin, and these adaptive differences are driven by distinct features of the climate and local environment, so that changes to contemporary conditions may disrupt locally adapted genotypes. For results based on localized environmental variables (Figures 2C, S12), patterns were difficult to discern given limited spatial coverage. However, RDA axis one generally contrasted the Bayfield Peninsula with other areas of the basin—adaptive diversity in this region was primarily shaped by poorer demographic population status but greater percentages of open mainstem river habitats (positive RDA scores). Relationships on RDA axis two generally contrasted easterly and westerly sites; adaptive diversity at westerly sites was largely driven by warmer summer air temperatures (positive RDA scores) whereas diversity at easterly sites was driven by greater groundwater input (negative RDA scores). For adaptive index derived from the climate dataset (Figure 3C), RDA axis one revealed contrasting regions that were intermixed across the landscape and differed in levels of snowfall and solar radiation, and in numbers of warm vs. cool degree days. In comparison, the southwestern portion of the Lake Superior basin was differentiated on RDA axis two. This region was generally characterized by greater levels of summer precipitation (negative RDA scores).

**FIGURE 4:**
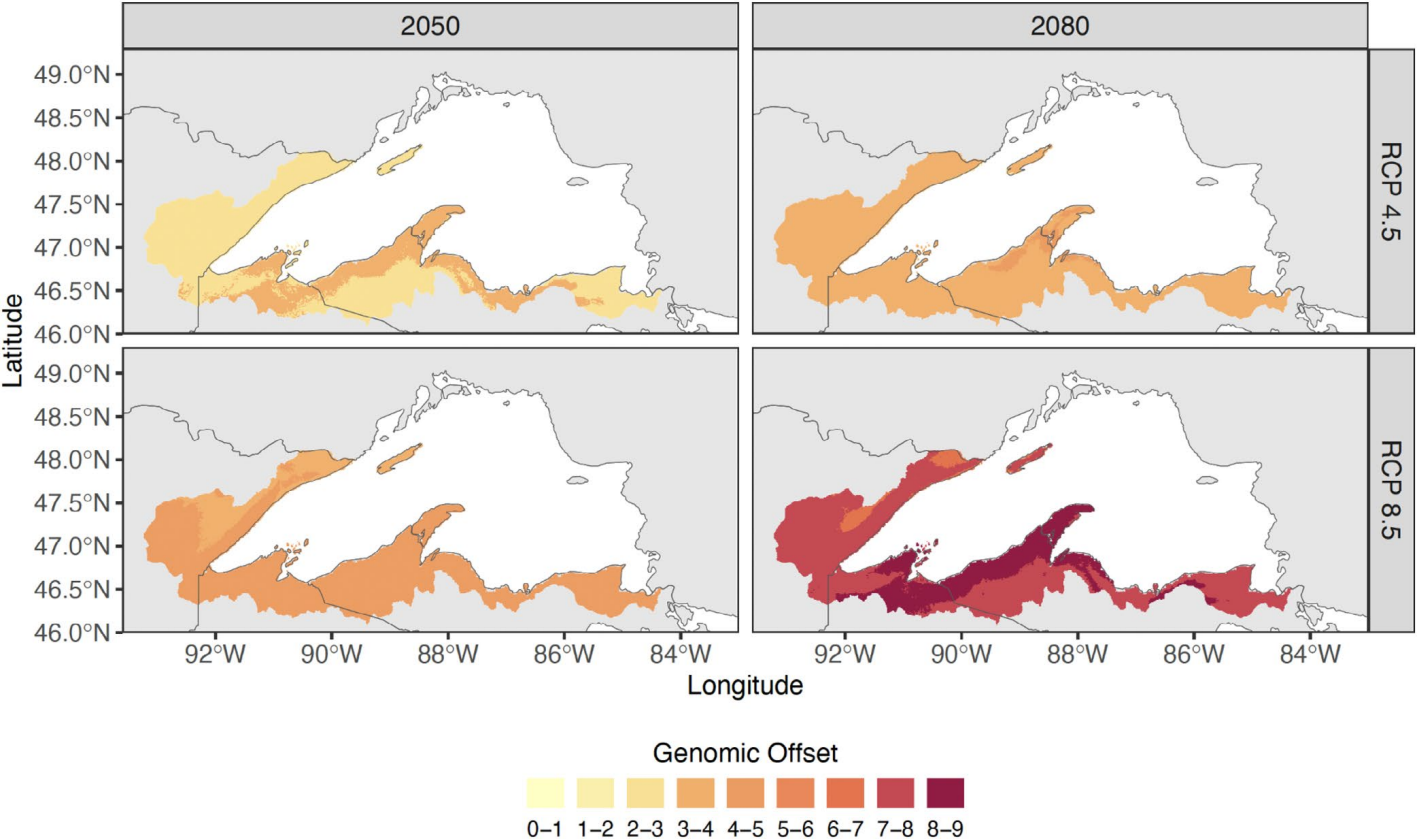
Future landscape based on genomic offset calculated from aeRDA performed using climate variables. Results are shown for the RCP4.5 and RCP8.5 emissions scenarios (rows) at the years 2050 and 2080 (columns).

A future landscape based on genomic offset revealed areas where brook trout may be most vulnerable to climate change. In this future landscape (Figure 4), which we estimated only for the climate dataset, the Keweenaw Peninsula and adjacent areas to the east and west exhibited greater genomic offset than other areas of the Lake Superior basin. This result indicates that brook trout inhabiting this region may require greater levels of adaptation to keep pace with changing climatic conditions compared to brook trout from elsewhere across the basin. In each of the projected climate scenarios we tested, brook trout across most of the Lake Superior basin exhibited comparatively intermediate levels of genomic offset and offset was greatest under the high emissions scenario (RCP8.5) for the year 2080.

## 4 Discussion

Information on environmental adaptation is urgently needed for conserving freshwater biodiversity given that many of these species are already impacted by diverse anthropogenic stressors, some of which are increasingly intensified by a changing climate. This information is critical for minimizing disruptions to locally adapted genetic diversity and for guiding active management interventions intended to facilitate targeted change–both approaches may be needed to support the resiliency and adaptive capacity of wild populations (Gaitán‐Espitia and Hobday 2021; Meek et al. 2023; Moore and Schindler 2022). Although there have been multiple studies focused on resolving population genetic relationships of brook trout native to the Lake Superior basin, to our knowledge our study is the first to explicitly consider environmental adaptation. Additionally, our study is among a small number of empirical studies so far that explore adaptation across a broad spatial extent and consider a rich array of environmental drivers, including local factors.

We distinguished a suite of SNPs that potentially form a genetic basis to adaptation in brook trout from the Lake Superior basin. We also identified variables that quantify impacts of dams and introduced species–pervasive features of the landscape in the Great Lakes and globally (Cooper et al. 2017; Simberloff et al. 2013)—as potentially important drivers of adaptive diversity, along with more commonly appreciated variables for air temperature and stream flow. Additionally, we identified locations where brook trout appear heterogeneously adapted to the landscape and where populations may face the greatest difficulty tracking climate change. Alarmingly, our genetically informed predictions are consistent with recent predictions based on habitat suitability modeling in the same geographic region (Yu et al. 2025), emphasizing the urgency of climate mitigation and adaptation planning. Broadly, our findings highlight the role that movement barriers play in shaping adaptive diversity in freshwater habitats and demonstrate the value of integrating system‐specific information into studies of environmental adaptation.

### 4.1 Environmental Adaptation in Brook Trout and Related Species

The relationships reported here highlight the multifaceted aspects of climate and local environment that potentially influence adaptation in freshwater species. A growing body of evidence from studies of wild trout and salmon implicate temperature and precipitation as major drivers of genetic adaptation (Andrews et al. 2023; Chen et al. 2018; Dallaire et al. 2021; Hecht et al. 2015; Meek et al. 2025; Micheletti et al. 2018; Tigano et al. 2024). In our study, two of the outlier SNPs identified using pRDA were also significant in the GO enrichment analysis; these SNPs were most strongly associated with variables for temperature (L_j_temp and L_temp; Table S3). Additionally, another outlier SNP—which was identified as an outlier in all four barrier datasets and the climate dataset—was identified as an expression quantitative trait locus associated with thermal stress response in a controlled experiment conducted using wild brook trout from New York (Meek et al. 2025). This SNP, which is located on chromosome two of the *Salvelinus* sp. genome, was most strongly correlated with mean annual precipitation (L_precip; Table S3; *r* = 0.11) in three of the barrier datasets and degree days above 18°C (DD18; Table S4; *r* = −0.11) in the climate dataset. Although it is difficult to disentangle the relative influences of precipitation and temperature on selection, these results strongly suggest this locus is involved in environmental adaptation within the Lake Superior basin and elsewhere across the native range.

The same climatic variables found to drive genetic adaptation, specifically temperature and precipitation, are the primary factors projected to drive drastic reductions in habitat available to brook trout in the future. Rising stream temperatures combined with altered flow conditions due to climate change are expected to reduce the stream habitat available to brook trout across the Great Lakes region (Mitro et al. 2019; Stewart et al. 2016; Yu et al. 2025), with losses of more than 60% predicted for Wisconsin streams (Mitro et al. 2019). Substantial losses to brook trout stream habitat are also predicted across the Midwestern and Northeastern United States, with most habitat loss centered on the Michigan Upper Peninsula and remaining areas of the southern Lake Superior basin (Yu et al. 2025). Coldwater lake habitats across the Midwest are also predicted to decrease by more than 60% and will have implications for coldwater species including trout (Hansen et al. 2022). Our work provides critically needed foundational knowledge for continued studies of environmental adaptation in brook trout from the Lake Superior basin, including in the context of climate change.

Studies of genetic adaptation offer a valuable complement to studies that employ non‐molecular methods to explore how climate and other environmental factors affect population dynamics. For example, strong associations between climate variation, particularly in temperature and precipitation, and variation in the abundance of younger age classes have been reported in previous studies of brook trout (Andrew et al. 2022; Bassar et al. 2016; Maitland and Latzka 2022). Similarly, temperature and precipitation have been shown to influence brook trout growth rates (Gallagher and Fraser 2024a; Xu et al. 2010) and spatial habitat use (Todd Petty et al. 2012). Findings from these and other studies suggest that climate can significantly affect the population dynamics of brook trout, which may in turn affect population‐level genetic diversity, for example through the differential survival of young‐of‐year brook trout under warming temperatures. Although context‐specific factors, including the evolutionary ancestry and adaptive capacity of populations (e.g., Mcdermid et al. 2012; Stitt et al. 2014) and fine‐scale differences in experienced environmental conditions (Gallagher and Fraser 2024a, 2024b; Valentine et al. 2024), also influence the actual response of populations to changing conditions, integrating insights across information sources is vital for identifying important themes and mitigating impacts to wild populations.

### 4.2 Importance of System‐Specific Information

Although the number of studies aimed at understanding environmental adaptation in wild populations is rapidly growing, these studies often lack system‐specific variables reflecting local conditions, so that important information about involved regions of the genome and associated drivers may be missed. For example, our variance partitioning results revealed that the localized environmental drivers explained 5% more genetic variation than the broad‐scale climate drivers. We also identified 73 additional outlier SNPs across the four datasets containing local environmental variables. These SNPs were not found using broad‐scale climate, resulting in an increase of more than 30% in the total number of outliers identified across all datasets. Integrating local environmental data is essential for producing more comprehensive insights into environmental adaptation and supporting the development of effective climate adaptation and mitigation strategies for wild populations.

By integrating localized environmental information, we identified system‐specific factors that potentially affect adaptation in brook trout native to the Lake Superior basin. For example, two variables for demographically informed population status (Table S3; U.S. Fish and Wildlife Service (USFWS) 2016) exhibited strong associations with the outlier SNPs identified from all four barrier datasets. This relationship potentially indicates that changes to population status may alter adaptive diversity, or that changes to adaptive diversity due to another factor may in turn affect population status. Similarly, a variable reflecting the presence of non‐native salmonids (Table S3), primarily coho salmon ( *Oncorhynchus kisutch* ), was strongly associated with outlier SNPs in two of the barrier datasets. Experimental studies report that juvenile coho salmon outcompete brook trout when resources are limited (Fausch and White 1986; Thornton et al. 2017), and surveys of wild populations in Lake Superior tributaries offer evidence for significant overlap in the diets of introduced coho and steelhead ( *O. mykiss* ) with brook trout (Howard 2013; but see Growe‐Raney 2016).

Resource competition between native and introduced salmonids can lead to spatial displacement and differential survival, which may have implications for adaptive diversity. For example, in systems where rainbow trout have been introduced, brook trout are often relegated to upstream reaches (Kanno et al. 2016) and occupy less preferable habitats (Thibault and Dodson 2013), which may shift the environmental and selective regimes they experience. As another example, coho salmon spawn later and hatch earlier than brook trout (Fausch and White 1986) so that in watersheds where these species are sympatric, spawning coho may dig up brook trout redds when preparing their own nests. Similarly, coho larvae can grow to larger sizes before brook trout larvae hatch (Fausch and White 1986), potentially allowing them to outcompete brook trout larvae for food. Although much work has focused on the genetic impacts of introgressive hybridization between native and introduced salmonids, considerably less work has focused on whether interspecific competition affects genetic adaptation.

### 4.3 Impact of Movement Barriers

Given the ubiquity of dams and other human‐made structures (e.g., culverts, weirs) that fragment streams and rivers, several studies have focused on understanding how these structures influence the spatial ecology and genetic diversity of aquatic populations (see reviews by Chan et al. 2025 and Zarri et al. 2022). Such knowledge is increasingly important as removing dams to restore connectivity is becoming a more realistic element of restoration programs for some native fishes (e.g., Duda et al. 2021; Fraik et al. 2021; Ramos and Ward 2023; Whittum et al. 2023), including due to the rising costs of maintaining ageing infrastructure (American Society for Civil Engineers (ASCE) 2021). However, the removal of some structures remains impractical (e.g., due to the large financial cost of removal) and in some contexts may be inappropriate (e.g., due to competing conservation or management issues; Walter et al. 2021). For example, many of the barriers constructed to limit dispersal of invasive sea lamprey ( *Petromyzon marinus* ) in the Great Lakes (Hrodey et al. 2021; Walter et al. 2021) play an important role in protecting native brook trout streams from invasion by sea lamprey, rainbow trout, and other species of Pacific salmon. Collectively, these factors highlight the need for more comprehensive information on the effects of movement barriers on aquatic ecosystems to guide management strategies aimed at minimizing negative impacts.

Here, variables reflecting movement barriers and their impacts on stream fragmentation were among the main drivers of putative adaptive diversity. In particular, a variable reflecting the total number of dams on a mainstem (variable 13 [TotalMainCnt]; Table S3) exhibited strong associations within every barrier dataset except the dataset for small dams, suggesting that large dams and waterfalls play especially important roles in shaping adaptation. These structures can severely alter stream conditions (flow, temperature, sedimentation) and shift the dynamics of lotic vs. lentic habitats (Cooper et al. 2017), which may be especially pronounced in waterways with more barriers, creating strong selective pressures. Our findings are consistent with previous studies that examined genetic connectivity of brook trout near waterfalls (D'Amelio and Wilson 2008; Kelson et al. 2015; Mamoozadeh et al. 2023; Scribner et al. 2012; Torterotot et al. 2014), which identified these structures as major drivers of genetic differentiation. Similarly, Timm et al. (2016) reported that genetic connectivity in brook trout is noticeably impacted by dams greater than 4 m in height, suggesting that lower profile dams may have comparatively small effects on genetic connectivity, consistent with our findings. Furthermore, we expect dams and other movement barriers to have a larger effect on genetic diversity in species with broad dispersal capabilities compared to species with limited dispersal (Day et al. 2024). Given this, it may be easier to disentangle the effects of barriers from other landscape features in broadly dispersing species (Day et al. 2024), and this may partially explain the large percentage of confounded variance in our variance partitioning results.

Our findings suggest that movement barriers are equally important drivers of adaptation as other commonly implicated climate variables. Population fragmentation due to dams and other structures can alter the selective regime experienced by aquatic populations, which may in turn elicit changes in adaptive variation (Zarri et al. 2022), though these changes likely also depend on factors including population size (Fraser et al. 2014). Altered selective regimes due to fragmentation affect the life history (Letcher et al. 2007), physiology (Hahlbeck et al. 2023), and other characteristics of isolated populations. Further, dams explain a large degree of variation in fish community structure, with this influence being as much or greater than other more commonly analyzed landscape features (Cooper et al. 2017; Wang, Infante, Lyons, et al. 2011), and may especially negatively affect coldwater species (Cooper et al. 2016). Collectively, our findings demonstrate that movement barriers, introduced species, and climate are important drivers of adaptation for brook trout in the Lake Superior basin.

### 4.4 Influence From Coaster Brook Trout

Migratory coaster brook trout were historically abundant across the Lake Superior basin, and their movements presumably had a substantial influence on the spatiotemporal distribution of genetic diversity in this region, an influence which likely continues in areas where coasters persist today. For example, straying coasters likely facilitate gene flow across streams (Elias et al. 2018), introducing genetic variation to stream resident populations whose evolutionary trajectories may otherwise be dominated by isolation and genetic drift. Such gene flow supplies neutral and adaptive genetic variation important for long‐term population resiliency and adaptive capacity. Given this, the loss of coaster brook trout from many parts of the Lake Superior basin has likely resulted in declining basin‐wide genetic diversity, which may be further exacerbated by dams and other movement barriers. Declines in genetic diversity may also be driven by a changing climate, as indicated by our estimates of genomic offset. Analogous studies which extend our work to include remnant coaster populations will be helpful to further understand the contributions of this life history form to contemporary genetic connectivity and climate vulnerability across the basin.

### 4.5 Analytical Context

Understanding the strengths and limitations of the analytical methods used to disentangle ecologically and evolutionarily important relationships in species central to conservation and management is important for deriving meaningful insights and refining informational needs. For example, interpreting results from genomic offset is difficult and depends on multiple factors, including system‐specific factors such as the relative influence of drift vs. selection on population dynamics (Lotterhos 2024), which is often poorly known. Current methods for calculating genomic offset also do not distinguish positive values that reflect genetic change needed to maintain fitness under future conditions from positive values that reflect an increase in fitness under future conditions (Lotterhos 2024). Additionally, our use of the 1961–1990 climate baseline predates our sampling, which largely occurred in 2011, so that absolute values of genomic offset may be slightly inflated. However, the relative spatial pattern revealed by our estimates of genomic offset offers a robust picture of differential climate risk. Despite these caveats, genomic offset provides similar or more accurate predictions about fitness under future conditions compared to analogous nongenetic methods (Lind et al. 2024). The genomic offset results presented here are consistent with predictions developed for brook trout in the Midwestern United States based on habitat modeling (Mitro et al. 2019; Stewart et al. 2016) and offer valuable information for prioritizing locations across the Lake Superior basin where populations may be especially vulnerable to climate change.

### 4.6 Implications for Conservation and Management

Understanding how populations are adapted to their local environment is vital for developing conservation and management plans that protect the capacity of populations to adapt to changing conditions (Hoffmann and Sgro 2011; Meek et al. 2023). For example, just as information derived from neutral genetic diversity is necessary to delimit management units comprising demographically independent groups of individuals, knowledge of adaptive diversity is needed to delimit adaptive groups that comprise management units similarly adapted to the local environment (Funk et al. 2012). Advances in our understanding of genetic adaptation have also propelled conversations about the role of active management interventions, such as assisted migration and genetic rescue (Chen et al. 2022; Fitzpatrick et al. 2023; Ralls et al. 2018; White, Rash, and Kazyak 2023), in conserving imperiled populations. For example, Kovach et al. (2022) identified populations of westslope cutthroat trout ( *O. clarkii lewisi* ) in need of genetic rescue using a dataset that included genotypes at expressed sequence tags. Similarly, there has been a long history of using translocations to rehabilitate and reestablish native populations of brook trout (Kanno et al. 2016; Smith et al. 2024; White, Johnson, et al. 2023). Our estimates of genomic offset help fisheries managers prioritize populations least capable of adapting to environmental change, while our adaptive index estimates are useful for identifying areas that support similarly adapted populations which may be used to enhance population resiliency or restore extirpated populations. For example, efforts to mitigate poor quality habitat, competition with non‐native salmonids, or other factors negatively impacting the health of native brook trout may be especially beneficial in areas such as the Keweenaw Peninsula, where genomic offset is expected to be comparatively high.

Recently available genetic forecasting capabilities, including estimates of genomic offset, offer a new horizon of insights to inform active management decisions. For example, Hung et al. (2023) calculated genomic offset based on putative adaptive diversity identified from critically endangered rosewood species (*Dalbergia* sp.). This information was used to make predictions about areas that may contain suitable seed sources for assisted translocation to focal restoration sites. Similarly, Lachmuth et al. (2023) combined genomic offset with ecological niche models to identify suitable donor and recipient populations to guide translocation activities while accounting for locally adapted genetic variation. The success of such translocation efforts may be further enhanced by integrating information on adaptive genetic diversity with other factors likely to affect the success of these efforts, including demographic structure and movement barriers (Seaborn et al. 2021). Collectively, the information generated in this and other analogous studies is vital for developing management plans that prioritize the most at‐risk populations, conserve locally adapted genotypes by mitigating specific aspects of the environment, and identify suitable source populations for active management interventions. Such efforts are particularly important for freshwater populations, including native populations of trout, given reduced dispersal opportunities and mounting human‐induced stressors.

## 5 Conclusions

Our study offers novel insights into genetic adaptation to broad‐scale climate and localized environment in brook trout native to the Lake Superior basin. These insights are critical for informing short‐ and long‐term management plans for brook trout, a species sensitive to the conditions affected by climate change (such as temperature and flow) and an array of other environmental stressors (such as habitat fragmentation). Importantly, we shed light on the role of lesser appreciated variables—dams and introduced species—in shaping adaptation in native populations of brook trout. These insights were made possible by leveraging both widely accessed climate data and locally derived environmental data, highlighting the vital importance of both information sources. Given the ubiquity of dams and introduced species in the Great Lakes, across the United States, and globally, our work highlights a need for further studies to assess how these factors shape environmental adaptation across aquatic biodiversity. Analogous work is also critical for brook trout native to other Great Lakes basins, where studies of population genetic relationships are limited, especially studies aimed at understanding environmental adaptation. Experimental work to further elucidate the role specific regions of the genome play in environmental adaptation will be an important complement to these studies. Collectively, insights from this work will provide important keys to unlock better restoration success for native species that ensure the resiliency and persistence of populations now and into the future.

## Funding

This work was supported by the US Fish and Wildlife Service, F17AC00774.

## Conflicts of Interest

The authors declare no conflicts of interest.

## Supporting information


**Data S1:** Supplemental_Methods.

**Figure S1:** Map depicting the sampling locations for the brook trout used to develop the RAD capture panel. Points reflect sampling sites within the Lake Superior basin listed in Table S1.
**Figure S2:** Pairwise genetic differentiation (Fst; Panel A) and genetic diversity metrics (inbreeding coefficient, Fis; observed heterozygosity, Ho; expected heterozygosity, He; Panel B) calculated for brook trout from the waterways sampled in this study. Both panels show box plots where larger black points represent potential outlier observations. Smaller gray points represent individual observations.
**Figure S3:** Results from permutation analyses to determine the effect of the number of variables assigned to a category on variance partitioning results. For each predictor category, points for estimates from 100 permutations are overlaid by a box plot summarizing estimates across permutations. Box plots are color‐coded as in Table 1. Results are based on the dataset inclusive of locally derived environmental variables for combined barrier types.
**Figure S4:** Results from RDA conducted using the dataset inclusive of locally derived environmental variables for large dams. Vectors are color‐coded as in Table 1, and numeric labels match the field numbers listed in Table S2.
**Figure S5:** Results from RDA conducted using the dataset inclusive of locally derived environmental variables for small dams. Vectors are color‐coded as in Table 1, and numeric labels match the field numbers listed in Table S2.
**Figure S6:** Results from RDA conducted using the dataset inclusive of locally derived environmental variables for waterfalls. Vectors are color‐coded as in Table 1, and numeric labels match the field numbers listed in Table S2.
**Figure S7:** Bar and matrix plots depicting the number of SNPs identified as outliers in results from pRDA. Results are shown for pRDA conducted using climate variables and for the datasets inclusive of locally derived environmental variables. For the latter, results are shown for each barrier type (large dams, small dams, waterfalls, combined barrier types). The total number of outlier SNPs identified from each dataset is shown in the horizontal bar plot at left. Sets of outlier SNPs identified from one or more datasets are shown in the vertical bar plot and matrix plot at right.
**Figure S8:** Manhattan plots showing locations within the Salvelinus sp. reference genome of outlier SNPs identified via pRDA analyses. Results are shown for pRDA conducted using climate variables and for the datasets inclusive of locally derived environmental variables. For the latter, results are shown for each barrier type (large dams, small dams, waterfalls, combined barriers). Scaffolds are depicted as chromosome 40.
**Figure S9:** Results from aeRDA conducted using the dataset inclusive of locally derived environmental variables for large dams. Vectors are color‐coded as in Table 1, and numeric labels match the field numbers listed in Table S2.
**Figure S10:** Results from aeRDA conducted using the dataset inclusive of locally derived environmental variables for small dams. Vectors are color‐coded as in Table 1, and numeric labels match the field numbers listed in Table S2.
**Figure S11:** Results from aeRDA conducted using the dataset inclusive of locally derived environmental variables for waterfalls. Vectors are color‐coded as in Table 1, and numeric labels match the field numbers listed in Table S2.
**Figure S12:** Contemporary landscapes based on adaptive index calculated from aeRDA conducted using the dataset inclusive of locally derived environmental variables. Results are shown for each barrier dataset. Pixels correspond with sampling sites within the Lake Superior basin. The location of the Bayfield Peninsula is indicated with asterisks.

**Table S1:** Sampling details for the wild‐caught and domestically raised brook trout analyzed in this study. Also shown are the numbers of brook trout retained in the quality filtered datasets used for RAD capture panel design and for genotype‐environment association analyses based on locally derived environmental data or broad‐scale climate data. The sampling sites listed here are displayed in Figures 1 and S1.
**Table S2:** Genomic datasets used for RAD capture panel design and genotype‐environment analyses. The quality filters applied to the individuals, SNPs, and genotypes comprising each dataset are shown. For the quality filtered datasets used in genotype‐environment analyses, the number of brook trout retained in the dataset inclusive of localized environmental variables (Local) relative to the dataset inclusive of broad scale climate variables (Broad) is shown.
**Table S3:** Details for the locally derived environmental variables evaluated in this study.
**Table S4:** Details for the climate variables evaluated in this study. These variables were all assigned to the climate category for variance partitioning. All of the variables listed here were sourced from Wang et al. 2016. Variable names listed in the AdaptWest database were retained.
**Table S5:** Pearson correlation coefficients for the full set of locally derived environmental variables and all barrier types. The field numbers listed here match those listed in Table S3.
**Table S6:** Pearson correlation coefficients for the full set of locally derived environmental variables and large dams. The field numbers listed here match those listed in Table S3.
**Table S7:** Pearson correlation coefficients for the full set of locally derived environmental variables and small dams. The field numbers listed here match those listed in Table S3.
**Table S8:** Pearson correlation coefficients for the full set of locally derived environmental variables and waterfalls. The field numbers listed here match those listed in Table S3.
**Table S9:** Pearson correlation coefficients for the final set of locally derived environmental variables and all barrier types. The field numbers listed here match those listed in Table S3.
**Table S10:** Pearson correlation coefficients for the final set of locally derived environmental variables and large dams. The field numbers listed here match those listed in Table S3.
**Table S11:** Pearson correlation coefficients for the final set of locally derived environmental variables and small dams. The field numbers listed here match those listed in Table S3.
**Table S12:** Pearson correlation coefficients for the final set of locally derived environmental variables and waterfalls. The field numbers listed here match those listed in Table S3.
**Table S13:** Pearson correlation coefficients for the full set of climate variables. The fields listed here match those listed in Table S4.
**Table S14:** Pearson correlation coefficients for the final set of climate variables. The fields listed here match those listed in Table S4.
**Table S15:** Results from variance partitioning conducted using locally derived environmental variables and large dams.
**Table S16:** Results from variance partitioning conducted using locally derived environmental variables and small dams.
**Table S17:** Results from variance partitioning conducted using locally derived environmental variables and waterfalls.
**Table S18:** GO terms enriched in regions of the genome that contained outlier SNPs identified via the pRDA conducted using locally derived environmental variables and all barriers. GO terms correspond with genes located within 10 kbp of outlier SNPs. Numbers of genes with GO annotations are shown for the reference dataset (No. Annotated Genes), outlier dataset (No. Significant Genes), and expected number (No. Expected Genes) based on a random distribution. A Wright‐Fisher correction was applied to raw *p*‐values (*p*‐value) and an alpha level of 0.01 was used to determine significance.

## Data Availability

Sequence data generated in this study are deposited in the NCBI Sequence Read Archive (PRJNA1458879).
