# Supplementary material for: Dams and Introduced Species Drive Patterns of Environmental Adaptation in an Iconic but Imperiled Coldwater Fish (Brook Trout, Salvelinus fontinalis)
Source: Evol Appl. 2026 May 3;19(5):e70239. doi: 10.1111/eva.70239 (PMC13136510; doi:10.1111/eva.70239)
Supplement: Supplementary file 1 — Data S1: Supplemental_Methods. [file EVA-19-e70239-s002.pdf]

SUPPLEMENTARY METHODS

Dams and introduced species drive patterns of environmental adaptation in an iconic but imperiled coldwater fish (brook trout, *Salvelinus fontinalis*)

Nadya Mamoozadeh[1,2*], Arthur Cooper[3], Henry Quinlan[4], Anna Varian[5], Dana Infante[3], & Mariah Meek[2,6]

[1]Department of Applied Ecology, North Carolina State University, 100 Eugene Brooks Avenue, Raleigh, NC 27695
[2]Department of Integrative Biology, Michigan State University, 288 Farm Lane, East Lansing, MI 48824
[3]Department of Fisheries and Wildlife, Michigan State University, 480 Wilson Road, East Lansing, MI 48824
[4]U.S. Fish and Wildlife Service, Ashland Fish and Wildlife Conservation Office, 2800 Lake Shore Drive, Ashland, WI 54806 (*retired*)
[5]Stantec, 2080 Wooddale Drive, Woodbury, MN 55125
[6]The Wilderness Society, 503 West Mendenhall Street, Bozeman, MT 59715

[*]Corresponding Author: Nadya Mamoozadeh (nrmamooz@ncsu.edu)

1. Field sampling and DNA isolation

We collected non-lethal fin tissue samples from brook trout (*Salvelinus fontinalis*) across the Lake Superior basin. In the U.S. portion of the basin, we sampled brook trout in 58 streams and rivers tributary to Lake Superior and at four locations within the lake during the years 2008–2017 (Table S1; Figure 1). The number of sites sampled within each of these waterways ranged from 1–17 (mean = 5 sites). Additionally, we analyzed brook trout from six sites across the Canadian portion of the Lake Superior basin (Table S1; Figure 1). Sampling of wild populations was primarily conducted via electrofishing in stream and shoreline habitats.

We also evaluated domestic brook trout from nine hatchery strains stocked within the Lake Superior basin (Table S1). Domestic brook trout were sampled from hatchery populations and used in this study to identify wild-caught brook trout of domestic origin. For the tissue samples described here, we isolated DNA using the magnetic bead-based protocol from Ali et al. (2016), then quantified DNA using Quant-iT PicoGreen assays (Thermo Fisher Scientific) to identify isolations suitable for high-throughput sequencing.

2. SNP discovery for RAD capture panel development

We created a new restriction site-associated DNA (RAD) capture panel (Ali et al., 2016) for Lake Superior basin brook trout to enable cost-effective genotyping of large numbers of individuals across a standardized set of SNPs. SNPs targeted in our RAD capture panel were discovered by conducting RAD sequencing (RADseq; Baird et al., 2008) on a subset of individuals selected to capture a wide range of genetic variation in brook trout across the Lake Superior basin. We prepared five RADseq libraries using wild-caught brook trout from the major waterways located within each HUC8 subwatershed along the U.S. portion of the Lake Superior basin (Table S1; Figure S1). These libraries also included wild brook trout from sites along the Canadian extent of Lake Superior and from the hatchery strains described above. We prepared RADseq libraries following the methods of Ali et al. (2016), except we used 120 ng of DNA for restriction enzyme digestion and we incubated ligation reactions for 12 hours. These libraries underwent PE150 sequencing on an Illumina HiSeq 4000 sequencing platform.

We used the data from our initial sequencing efforts to identify SNPs to target in a RAD capture panel. We demultiplexed reads using the process_radtags module of Stacks (Catchen, Amores, Hohenlohe, Cresko, & Postlethwait, 2011; Rochette, Rivera-Colón, & Catchen, 2019) and options to tolerate a barcode mismatch of 1 bp and to discard reads with uncalled bases or low quality scores. Next, we mapped reads to the *Salvelinus sp.* reference genome (GCF_002910315; Christensen et al., 2018; but see Christensen et al., 2021) using the BWA MEM algorithm implemented in BWA (Li, 2013). We then used SAMtools (Li et al., 2009) to quality filter read alignments by removing unmapped reads, secondary and supplementary alignments, and alignments with quality scores < 30. Finally, we used Stacks v2.1 to identify SNPs from read alignments using the gstacks module, then exported genotypes as a VCF file using the populations module.

We performed quality filtering to produce a SNP dataset limited to high quality genotypes. We used VCFtools v0.1.15 (Danecek et al., 2011) to exclude SNPs missing > 80% of genotypes followed by individuals missing ≥ 90% of genotypes. We also excluded genotypes with read depths < 7 and quality scores < 30 (Table S2). We then iteratively removed loci missing > 50% of genotypes and individuals missing ≥ 50% of genotypes. Finally, we used HDplot (McKinney, Waples, Seeb, & Seeb, 2017) to identify SNPs likely located within duplicated regions of the brook trout genome. At this stage, we did not include a filter to remove

SNPs with low minor allele frequencies. Brook trout is a highly structured species with large degrees of population structure possible across small geographic distances (Mamoozadeh et al. 2023a, 2023b; Ferchaud et al., 2020). Because we sequenced a small number of brook trout per sampling site, removing SNPs that exhibited low minor allele frequencies in our dataset would unnecessarily exclude loci informative of population structure. Further, the other filters we employed during quality filtering targeted the removal of unreliable genotype calls.

The quality filtered dataset we used to design RAD capture baits contained genotypes for 290 brook trout at 326,010 SNPs (Table S2). This dataset included 248 wild-caught brook trout from 63 waterways across the Lake Superior basin (n = 1–10 [mean = 4 individuals] per waterway; Table S1). Additionally, this dataset included 42 domestically raised brook trout from nine hatchery strains (n = 2–7 [mean = 5] individuals per strain; Table S1). We used this dataset for RAD capture panel design.

3. Design of RAD capture baits

Baits used to target SNPs in our RAD capture panel were designed and manufactured by Arbor BioSciences (Ann Arbor, Michigan; www.arborbiosci.com) using myBaits v4 chemistry. Baits were designed from the contig sequences for the SNPs retained in our quality filtered dataset. Because baits are 80 bp long and are centered on the target SNP, SNPs within 40 bp of the 3' end of each contig were excluded from the design process. We also excluded SNPs located within repetitive regions of the *Salvelinus sp.* reference genome or located on unassembled scaffolds. For remaining SNPs, the SNP closest to the 5' end of each contig was used for bait design. We designed two baits per SNP, where baits targeted one of two alleles for the focal SNP to ensure high capture efficiency for each locus. For baits that spanned seven or eight total SNPs, additional baits were designed that incorporated variable sites for each possible allele. This was done to maintain capture efficiency for baits targeting regions of the genome with greater variability. Baits that spanned nine or more total SNPs were expected to exhibit poor capture efficiency and were thus excluded. We also excluded baits with suboptimal melting temperatures or that produced matches to off-target species in BLAST search results. Our final RAD capture panel comprised 59,013 baits targeting 33,309 SNPs corresponding with 25,639 contigs and distributed throughout the brook trout genome.

4. Validation of RAD capture panel performance

We tested the performance of our RAD capture panel by sequencing a capture reaction that included both previously and newly genotyped individuals. We prepared three new RADseq libraries as described above, then pooled 100 ng from each of these libraries with 100 ng from a library sequenced during the SNP discovery stage prior to bait design. We included a previously sequenced library in this pool so that we could assess the consistency of genotypes derived from RAD capture compared to directly sequencing a RADseq library. We used this library pool as template in a capture reaction performed following the Arbor BioSciences myBaits v4 manual (version 4.01 from April 2018). The resulting product then underwent 150 bp PE sequencing on an Illumina HiSeq X.

We used resulting sequence data to assess the performance of our RAD capture panel. We demultiplexed reads as described above, then evaluated whether the regions of the genome targeted by our RAD capture panel were recovered in our test run. We used BWA to map reads to the bait sequences comprising our RAD capture panel. The percentage of reads from each library that mapped to the bait sequences ranged from 73.52–81.61% (mean across libraries =

79.19%). We also compared the contigs used to design the capture baits with contigs built from our test data. To do this, we first mapped reads to the *Salvelinus sp.* reference genome, then performed quality filtering of read alignments and SNP calling as described above. We then used the intersect tool from bedtools (Quinlan & Hall, 2010) to identify contigs represented in both datasets. These results indicated that 97.24% of the contigs used for bait design were recovered in our test run. Finally, we assessed the consistency of genotypes for the library that was sequenced during the initial SNP discovery stage and sequenced again to test our RAD capture panel. We used VCFtools to compare VCF files between sequencing runs and determined that genotypes were consistent between genotyping methods. Collectively, the results described here indicated that our RAD capture panel was working as expected and ready for larger scale use.

5. High-throughput RAD capture genotyping

We used our validated RAD capture panel to perform high-throughput genotyping of brook trout from across the Lake Superior basin. We prepared 29 RADseq libraries that comprised both wild-caught and domestic brook trout; samples of brook trout that sequenced poorly in earlier stages of this study were included in the libraries prepared here. We used these RADseq libraries as template in five capture reactions, where six libraries were pooled in equal concentration for each reaction, then we used reaction products for 150 PE sequencing on an Illumina HiSeq X.

Resulting sequence data were used to identify SNPs and produce high quality genotypes for brook trout across the Lake Superior basin. We demultiplexed reads and mapped them to the *Salvelinus sp.* genome as described above. We then combined these data with data generated during earlier stages of this study and called SNPs using Stacks. We used VCFtools to perform quality filtering of the resulting VCF file by excluding SNPs missing > 20% of genotypes followed by individuals missing ≥ 70% of genotypes (Table S2). We then removed genotypes with quality scores < 30 and read depths < 5. We excluded genotypes with allele balance either > 0.80 or < 0.20 using jvarkit (Lindenbaum, 2015). We then used VCFtools to remove loci with a minor allele count < 3 followed by loci missing > 20% of genotypes and individuals missing ≥ 30% of genotypes. We removed paralogous SNPs by using HDplot to identify loci exhibiting excessive heterozygosity or read ratio deviations. We then excluded loci rendered monomorphic by the filtering process and retained a single SNP per RAD locus to reduce the level of linkage among loci; we did this by retaining the SNP that exhibited the highest minor allele frequency.

While the quality filtered dataset produced at this stage was useful for exploring population-level genetic relationships (results not shown), we performed additional filters to produce a dataset reliable for the genotype-environment analyses central to this study. We excluded brook trout of domestic origin and that were sampled from locations where landscape data were unavailable (Table S2). We then removed SNPs rendered monomorphic by the filtering process and excluded loci with minor allele frequencies < 1% to reduce bias from rare alleles in genotype-environment association analyses. Our final quality filtered SNP dataset comprised 3,297 SNPs genotyped at 2,251 individuals in the dataset inclusive of localized environmental variables and 2,229 individuals in the dataset based on broad-scale climate variables (Table S2). These brook trout were collected from 55 waterways and were identical between datasets, except for two sites where brook trout were not retained in the climate dataset. One of these sites was the only site sampled at the waterway (Omans Creek, Michigan, USA) so that only 54 waterways were represented in the climate dataset. The quality filtered datasets were used for the series of genotype-environment association analyses described in the main text.

REFERENCES

Ali, O., O'Rourke, S. M., Amish, S. J., Meek, M. H., Luikart, G., Jeffres, C., & Miller, M. R. (2016). RAD Capture (Rapture): Flexible and efficient sequence-based genotyping. *Genetics*, *202*, 389–400. https://doi.org/http://dx.doi.org/10.1101/028837

Baird, N. a, Etter, P. D., Atwood, T. S., Currey, M. C., Shiver, A. L., Lewis, Z. a, … Johnson, E. a. (2008). Rapid SNP discovery and genetic mapping using sequenced RAD markers. *PloS One*, *3*(10), e3376. https://doi.org/10.1371/journal.pone.0003376

Catchen, J. M., Amores, A., Hohenlohe, P., Cresko, W., & Postlethwait, J. H. (2011). Stacks: building and genotyping Loci de novo from short-read sequences. *G3: Genes, Genomes, Genetics*, *1*, 171–182. https://doi.org/10.1534/g3.111.000240

Catchen, J. M., Hohenlohe, P. A., Bassham, S., Amores, A., & Cresko, W. (2013). Stacks: an analysis tool set for population genomics. *Molecular Ecology*, *22*(11), 3124–3140. https://doi.org/10.1111/mec.12354.Stacks

Christensen, K. A., Rondeau, E. B., Minkley, D. R., Leong, J. S., Nugent, C. M., Danzmann, R. G., … Koop, B. F. (2021). Retraction: The Arctic charr (Salvelinus alpinus) genome and transcriptome assembly (PLoS ONE (2018) 13: 9 (e0204076) DOI: 10.1371/journal.pone.0204076). *PLoS ONE*, *16*(2), e0247083. https://doi.org/10.1371/journal.pone.0247083

Christensen, K. A., Rondeau, E. B., Minkley, D. R., Leong, J. S., Nugent, C. M., Danzmann, R. G., … Koop, F. (2018). The Arctic charr (Salvelinus alpinus) genome and transcriptome assembly. *PLoS ONE*, *13*(9), e0204076.

Danecek, P., Auton, A., Abecasis, G., Albers, C. a., Banks, E., DePristo, M. a., … Durbin, R. (2011). The variant call format and VCFtools. *Bioinformatics*, *27*(15), 2156–2158. https://doi.org/10.1093/bioinformatics/btr330

Ferchaud, A.-L., Leitwein, M., Laporte, M., Boivin-Delisle, D., Bougas, B., Hernandez, C., … Bernatchez, L. (2020). Adaptive and maladaptive genetic diversity in small populations; insights from the Brook Charr (Salvelinus fontinalis) case study. *Molecular Ecology*. https://doi.org/10.1101/660621

Gruber, B., Unmack, P. J., Berry, O. F., & Georges, A. (2018). DARTR: An R package to facilitate analysis of SNP data generated from reduced representation genome sequencing. *Molecular Ecology Resources*, *18*, 1–9. https://doi.org/10.1111/1755-0998.12745

Li, H. (2013). Aligning sequence reads, clone sequences and assembly contigs with BWA-MEM. *ArXiv Preprint*. https://doi.org/arXiv:1303.3997 [q-bio.GN]

Li, H., Handsaker, B., Wysoker, A., Fennell, T., Ruan, J., Homer, N., … Durbin, R. (2009). The Sequence Alignment/Map format and SAMtools. *Bioinformatics*, *25*(16), 2078–2079. https://doi.org/10.1093/bioinformatics/btp352

Lindenbaum, P. (2015). JVarkit: java-based utilities for Bioinformatics. *Figshare*, 2–5. https://doi.org/10.6084/m9.figshare.1425030.v1

McKinney, G. J., Waples, R. K., Seeb, L. W., & Seeb, J. E. (2017). Paralogs are revealed by proportion of heterozygotes and deviations in read ratios in genotyping-by-sequencing data from natural populations. *Molecular Ecology Resources*, *17*, 656–669. https://doi.org/10.1111/1755-0998.12613

Quinlan, A. R., & Hall, I. M. (2010). BEDTools: A flexible suite of utilities for comparing genomic features. *Bioinformatics*, *26*(6), 841–842. https://doi.org/10.1093/bioinformatics/btq033

Rochette, N. C., & Catchen, J. M. (2017). Deriving genotypes from RAD-seq short-read data

using Stacks. *Nature Protocols*, *12*(12), 2640–2659. https://doi.org/10.1038/nprot.2017.123
Rochette, N. C., Rivera-Colón, A. G., & Catchen, J. M. (2019). Stacks 2: Analytical methods for paired-end sequencing improve RADseq-based population genomics. *Molecular Ecology*, *28*, 4737–4754. https://doi.org/10.1111/mec.15253
