# Supplementary material for: Dams and Introduced Species Drive Patterns of Environmental Adaptation in an Iconic but Imperiled Coldwater Fish (Brook Trout, Salvelinus fontinalis)
Source: Evol Appl. 2026 May 3;19(5):e70239. doi: 10.1111/eva.70239 (PMC13136510; doi:10.1111/eva.70239)
Supplement: Supplementary file 2 — Figure S1: Map depicting the sampling locations for the brook trout used to develop the RAD capture panel. Points reflect sampling sites within the Lake Superior basin listed in Table S1. Figure S2: Pairwise genetic differentiation (Fst; Panel A) and genetic diversity metrics (inbreeding coefficient, Fis; observed heterozygosity, Ho; expected heterozygosity, He; Panel B) calculated for brook trout from the waterways sampled in this study. Both panels show box plots where larger black points represent potential outlier observations. Smaller gray points represent individual observations. Figure S3: Results from permutation analyses to determine the effect of the number of variables assigned to a category on variance partitioning results. For each predictor category, points for estimates from 100 permutations are overlaid by a box plot summarizing estimates across permutations. Box plots are color‐coded as in Table 1. Results are based on the dataset inclusive of locally derived environmental variables for combined barrier types. Figure S4: Results from RDA conducted using the dataset inclusive of locally derived environmental variables for large dams. Vectors are color‐coded as in Table 1, and numeric labels match the field numbers listed in Table S2. Figure S5: Results from RDA conducted using the dataset inclusive of locally derived environmental variables for small dams. Vectors are color‐coded as in Table 1, and numeric labels match the field numbers listed in Table S2. Figure S6: Results from RDA conducted using the dataset inclusive of locally derived environmental variables for waterfalls. Vectors are color‐coded as in Table 1, and numeric labels match the field numbers listed in Table S2. Figure S7: Bar and matrix plots depicting the number of SNPs identified as outliers in results from pRDA. Results are shown for pRDA conducted using climate variables and for the datasets inclusive of locally derived environmental variables. For the latter, results are shown [file EVA-19-e70239-s001.pdf]

SUPPLEMENTARY FIGURES

Dams and introduced species drive patterns of environmental adaptation in an iconic but imperiled coldwater fish (brook trout, *Salvelinus fontinalis*)

Nadya Mamoozadeh[1,2*], Arthur Cooper[3], Henry Quinlan[4], Anna Varian[5], Dana Infante[3], & Mariah Meek[2,6]

[1]Department of Applied Ecology, North Carolina State University, 100 Eugene Brooks Avenue, Raleigh, NC 27695
[2]Department of Integrative Biology, Michigan State University, 288 Farm Lane, East Lansing, MI 48824
[3]Department of Fisheries and Wildlife, Michigan State University, 480 Wilson Road, East Lansing, MI 48824
[4]U.S. Fish and Wildlife Service, Ashland Fish and Wildlife Conservation Office, 2800 Lake Shore Drive, Ashland, WI 54806 (*retired*)
[5]Stantec, 2080 Wooddale Drive, Woodbury, MN 55125
[6]The Wilderness Society, 503 West Mendenhall Street, Bozeman, MT 59715

[*]Corresponding Author: Nadya Mamoozadeh (nrmamooz@ncsu.edu)

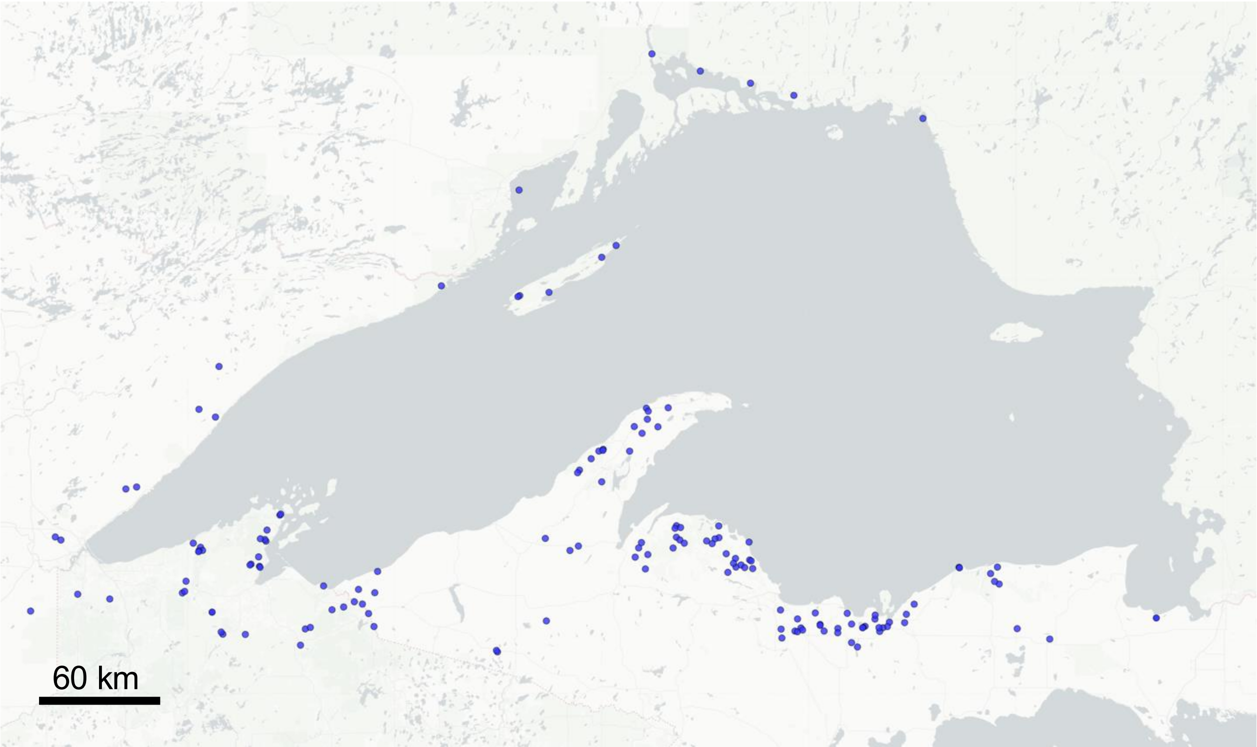

Figure S1. Map depicting the sampling locations for the brook trout used to develop the RAD capture panel. Points reflect sampling sites within the Lake Superior basin listed in Table S1.

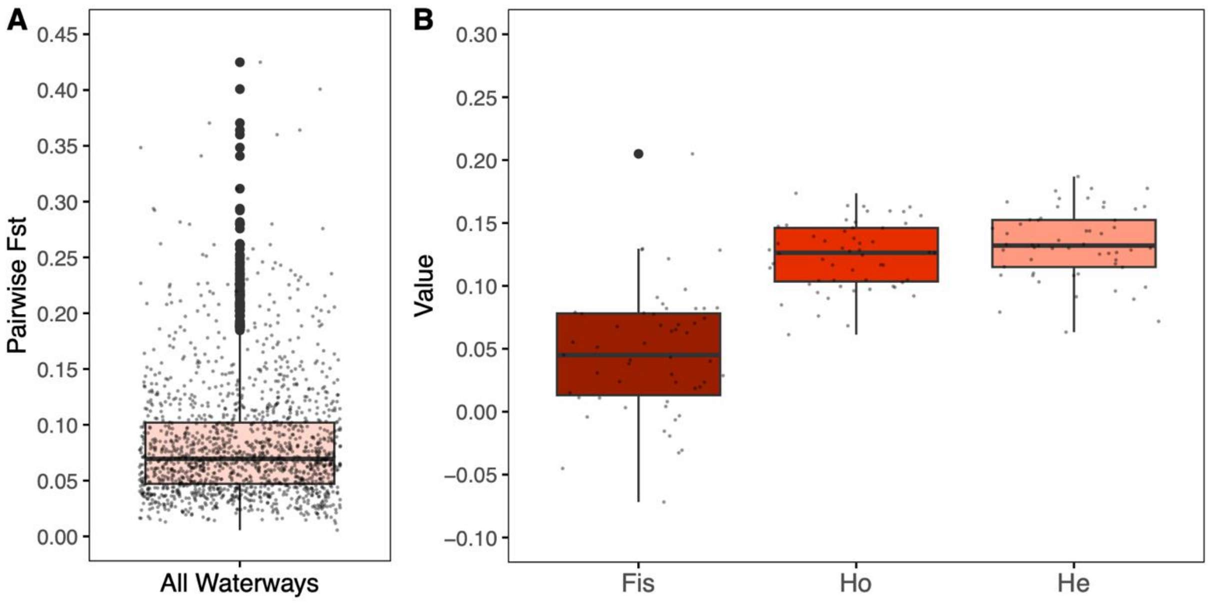

Figure S2. Pairwise genetic differentiation (Fst; Panel A) and genetic diversity metrics (inbreeding coefficient, Fis; observed heterozygosity, Ho; expected heterozygosity, He; Panel B) calculated for brook trout from the waterways sampled in this study. Both panels show box plots where larger black points represent potential outlier observations. Smaller gray points represent individual observations.

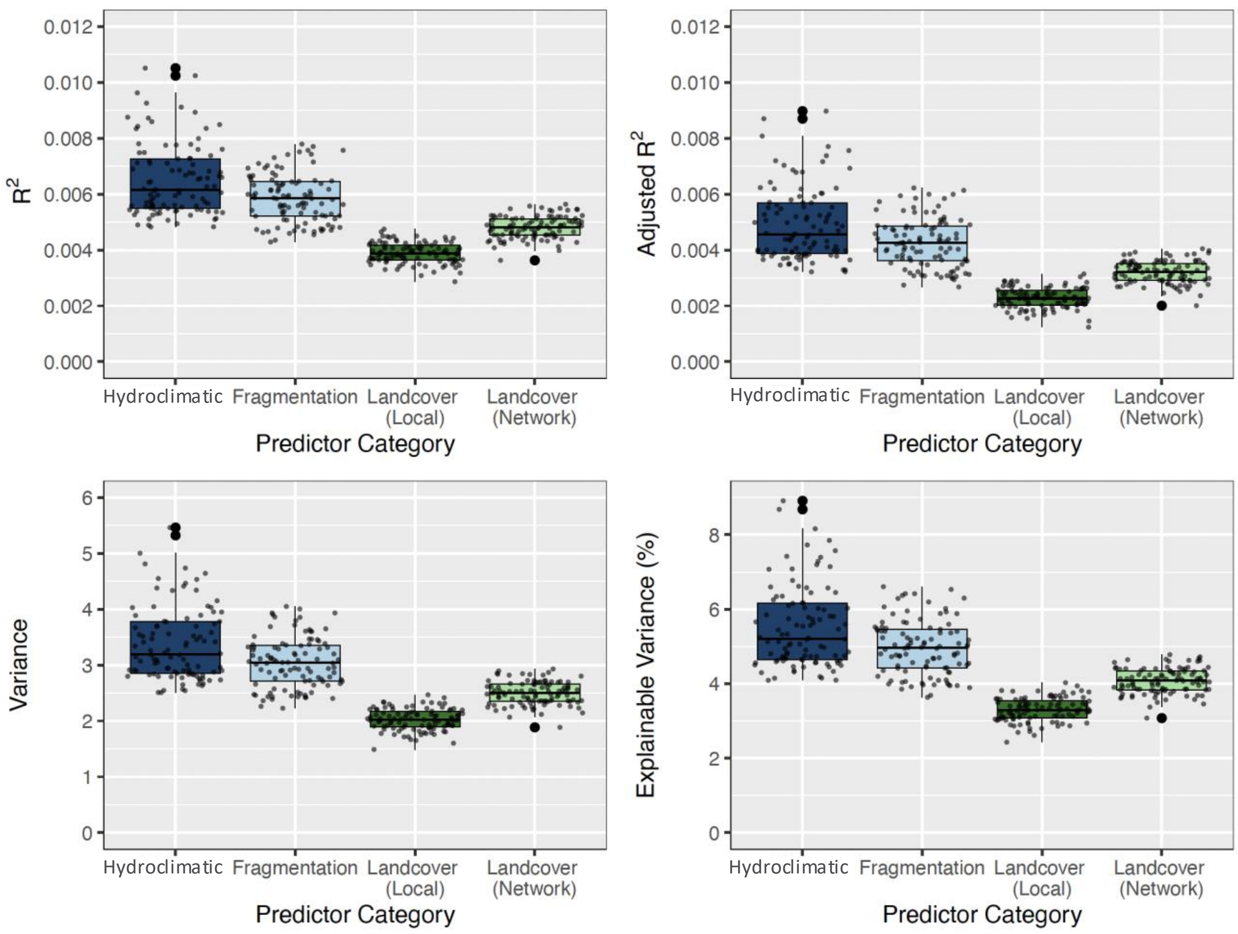

Figure S3. Results from permutation analyses to determine the effect of the number of variables assigned to a category on variance partitioning results. For each predictor category, points for estimates from 100 permutations are overlaid by a box plot summarizing estimates across permutations. Box plots are color-coded as in Table 1. Results are based on the dataset inclusive of locally derived environmental variables for combined barrier types.

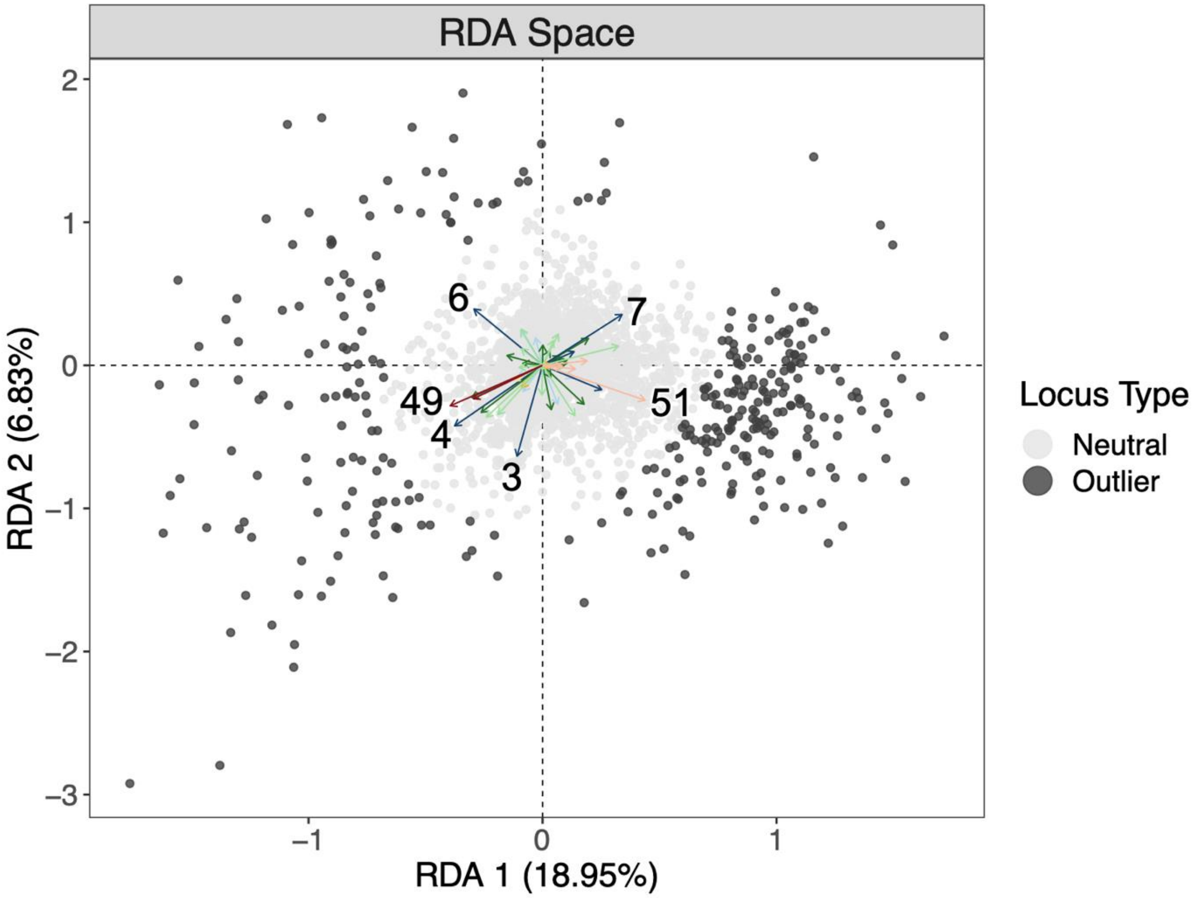

Figure S4. Results from RDA conducted using the dataset inclusive of locally derived environmental variables for large dams. Vectors are color-coded as in Table 1, and numeric labels match the field numbers listed in Table S2.

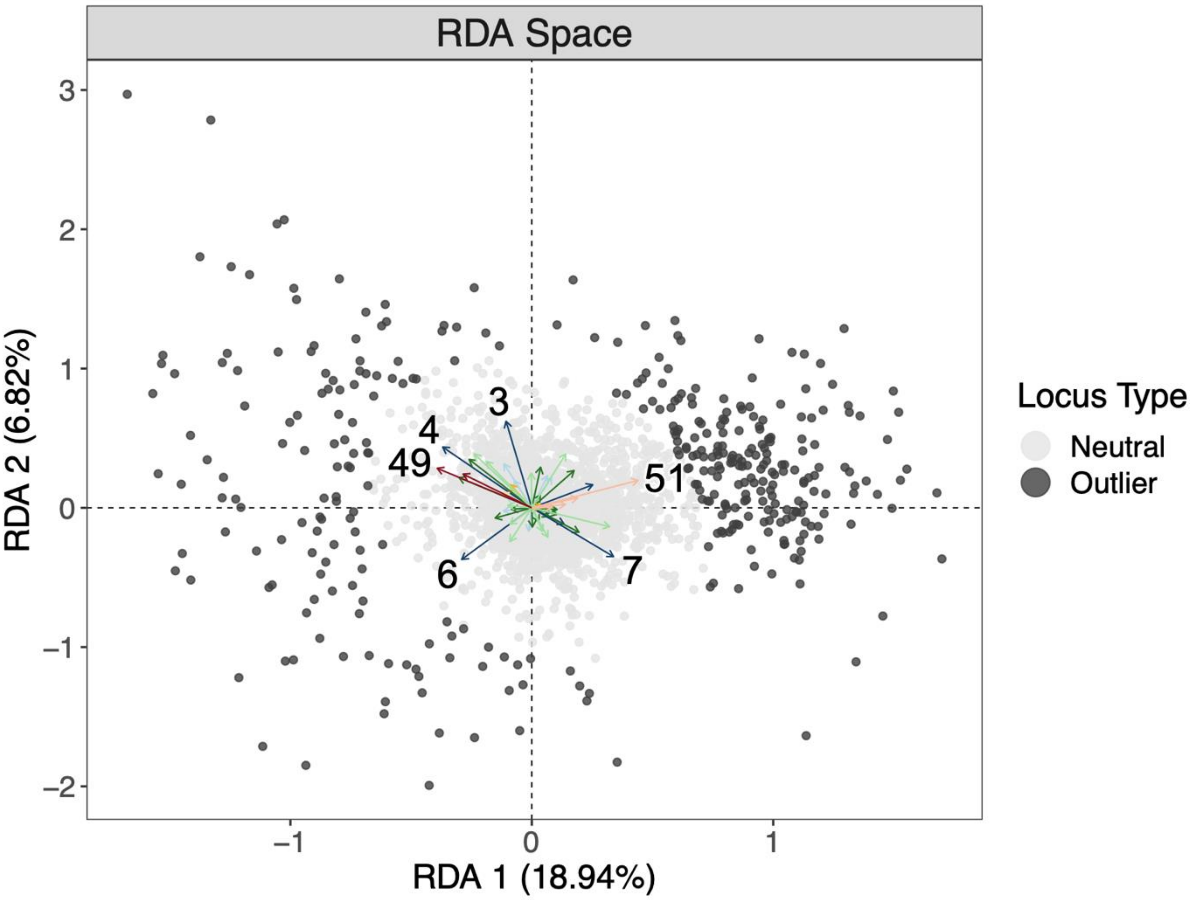

Figure S5. Results from RDA conducted using the dataset inclusive of locally derived environmental variables for small dams. Vectors are color-coded as in Table 1, and numeric labels match the field numbers listed in Table S2.

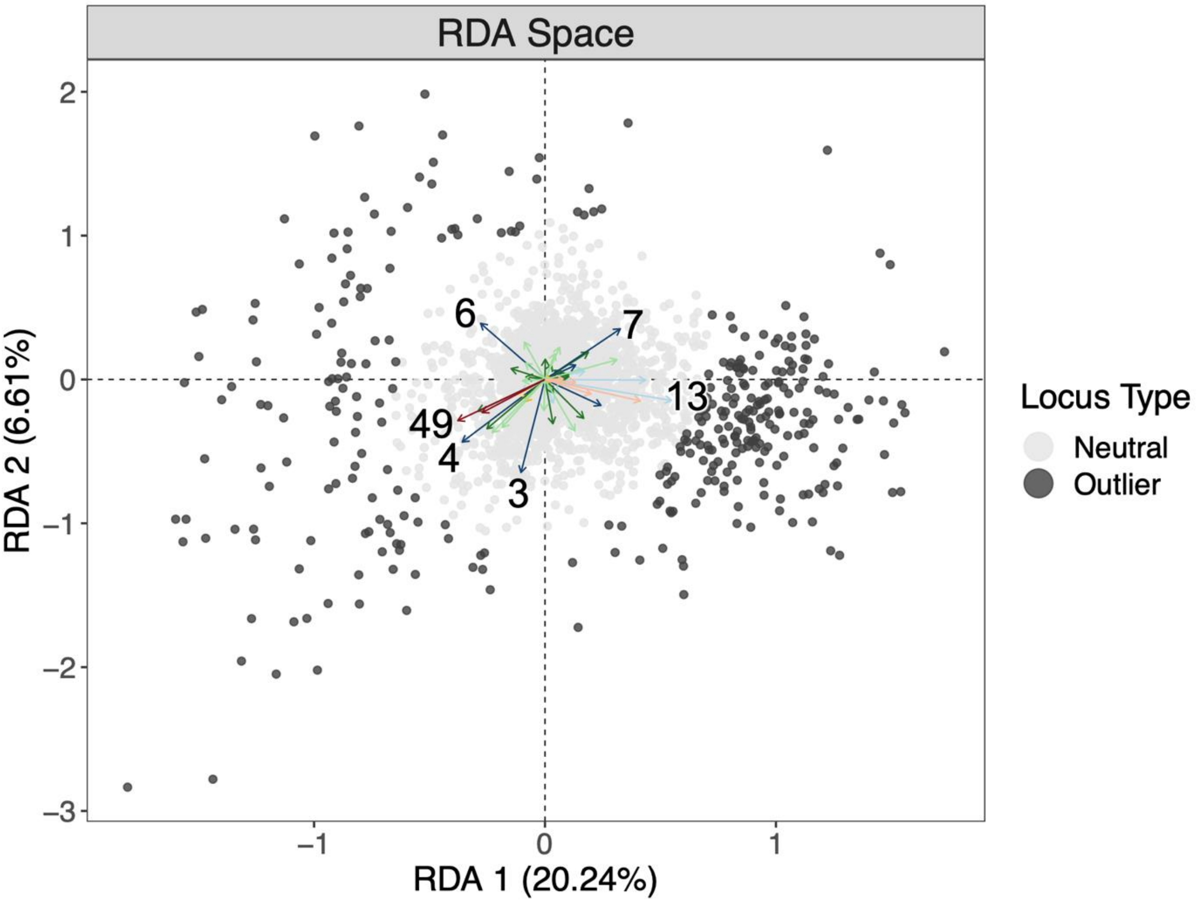

Figure S6. Results from RDA conducted using the dataset inclusive of locally derived environmental variables for waterfalls. Vectors are color-coded as in Table 1, and numeric labels match the field numbers listed in Table S2.

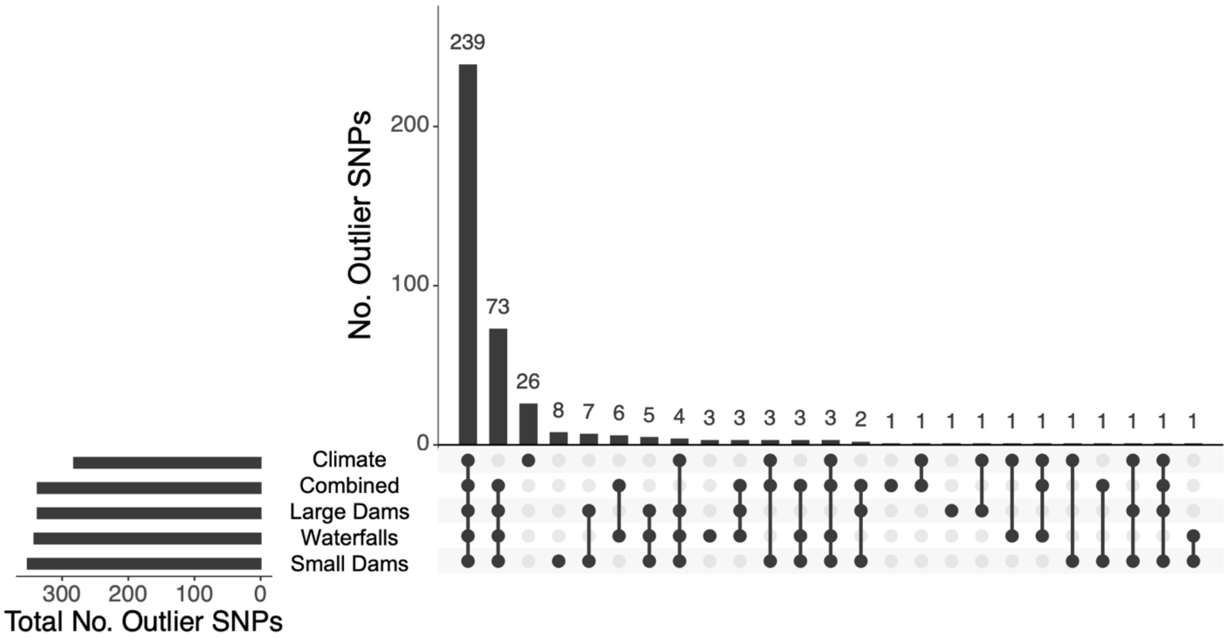

Figure S7. Bar and matrix plots depicting the number of SNPs identified as outliers in results from pRDA. Results are shown for pRDA conducted using climate variables and for the datasets inclusive of locally derived environmental variables. For the latter, results are shown for each barrier type (large dams, small dams, waterfalls, combined barrier types). The total number of outlier SNPs identified from each dataset is shown in the horizontal bar plot at left. Sets of outlier SNPs identified from one or more datasets are shown in the vertical bar plot and matrix plot at right.

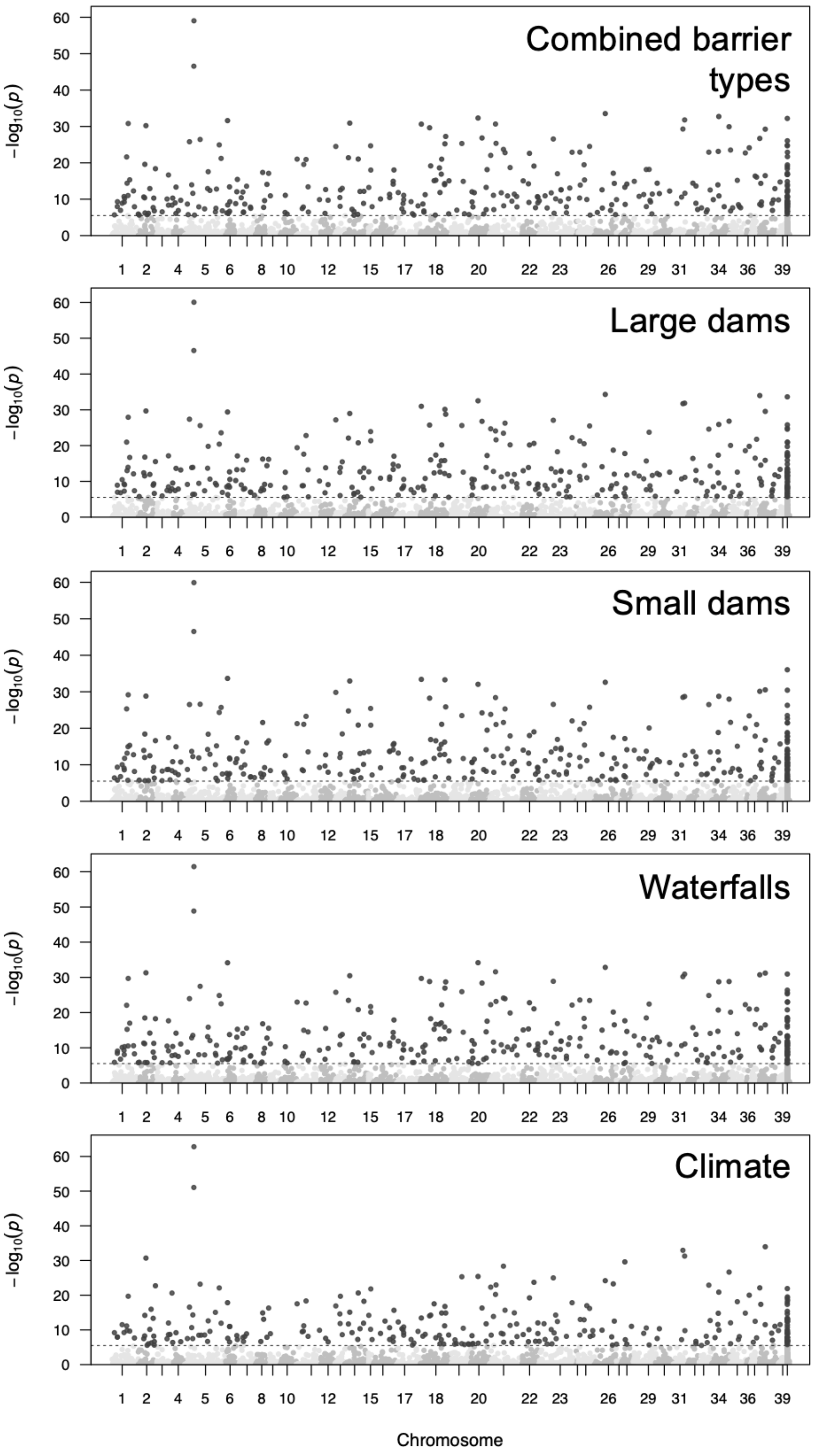

Figure S8. Manhattan plots showing locations within the *Salvelinus sp.* reference genome of outlier SNPs identified via pRDA analyses. Results are shown for pRDA conducted using climate variables and for the datasets inclusive of locally derived environmental variables. For the latter, results are shown for each barrier type (large dams, small dams, waterfalls, combined barriers). Scaffolds are depicted as chromosome 40.

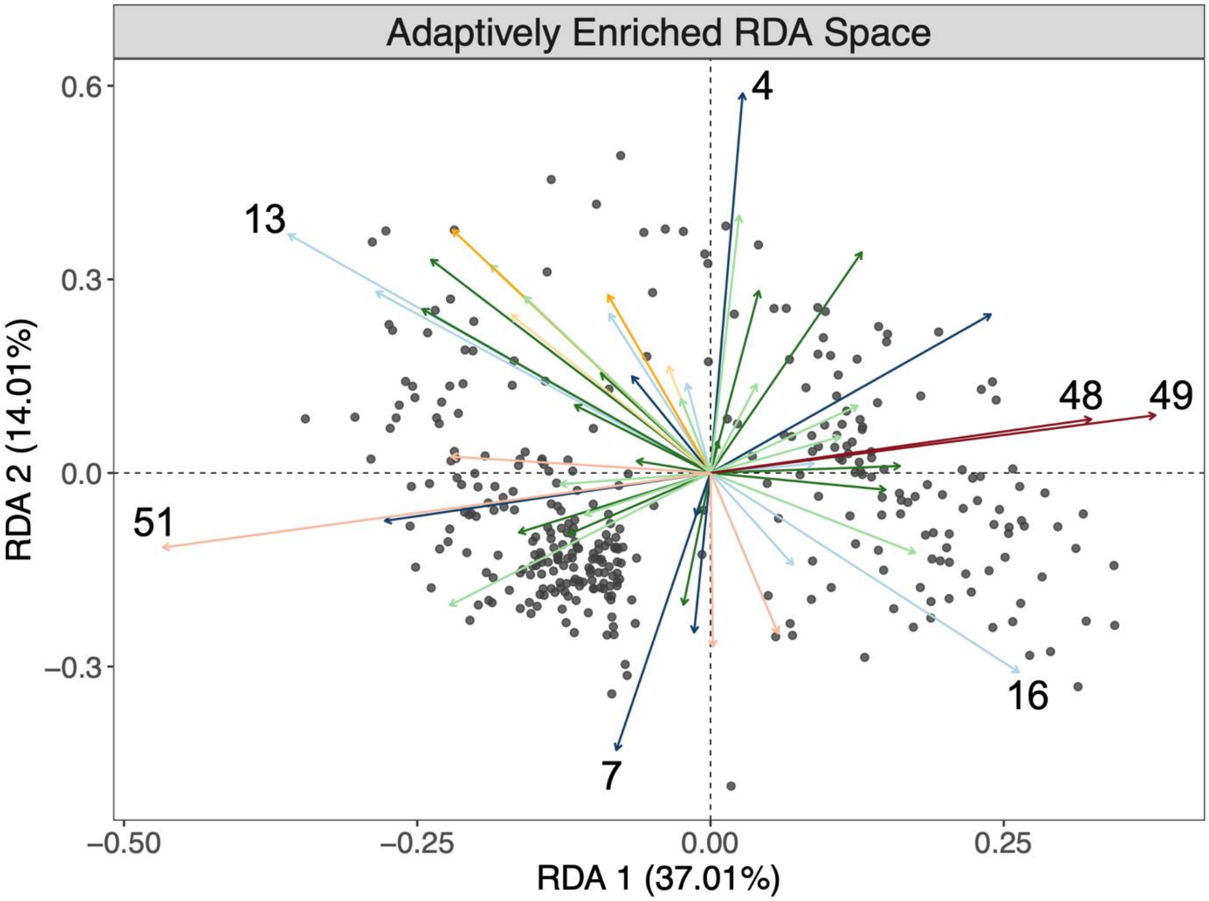

Figure S9. Results from aeRDA conducted using the dataset inclusive of locally derived environmental variables for large dams. Vectors are color-coded as in Table 1, and numeric labels match the field numbers listed in Table S2.

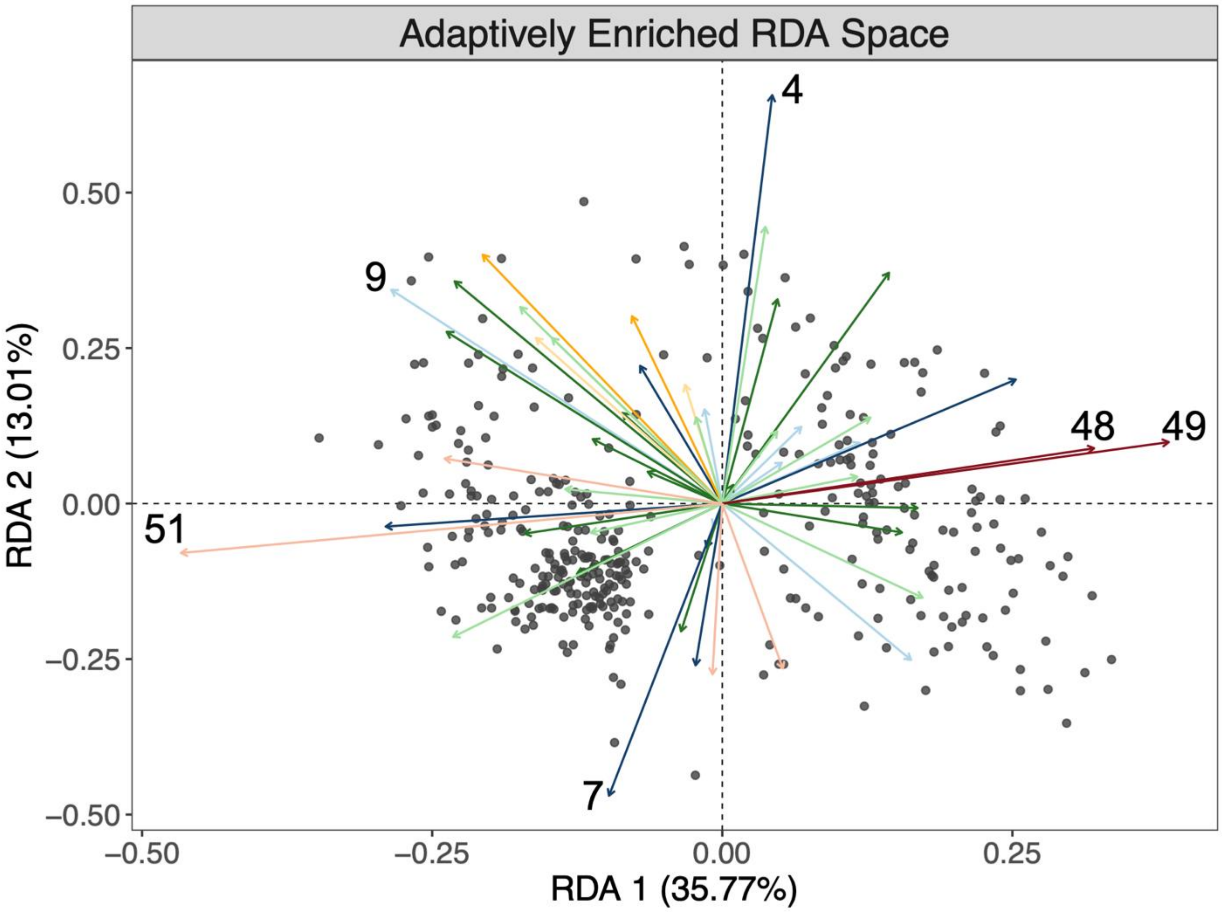

Figure S10. Results from aeRDA conducted using the dataset inclusive of locally derived environmental variables for small dams. Vectors are color-coded as in Table 1, and numeric labels match the field numbers listed in Table S2.

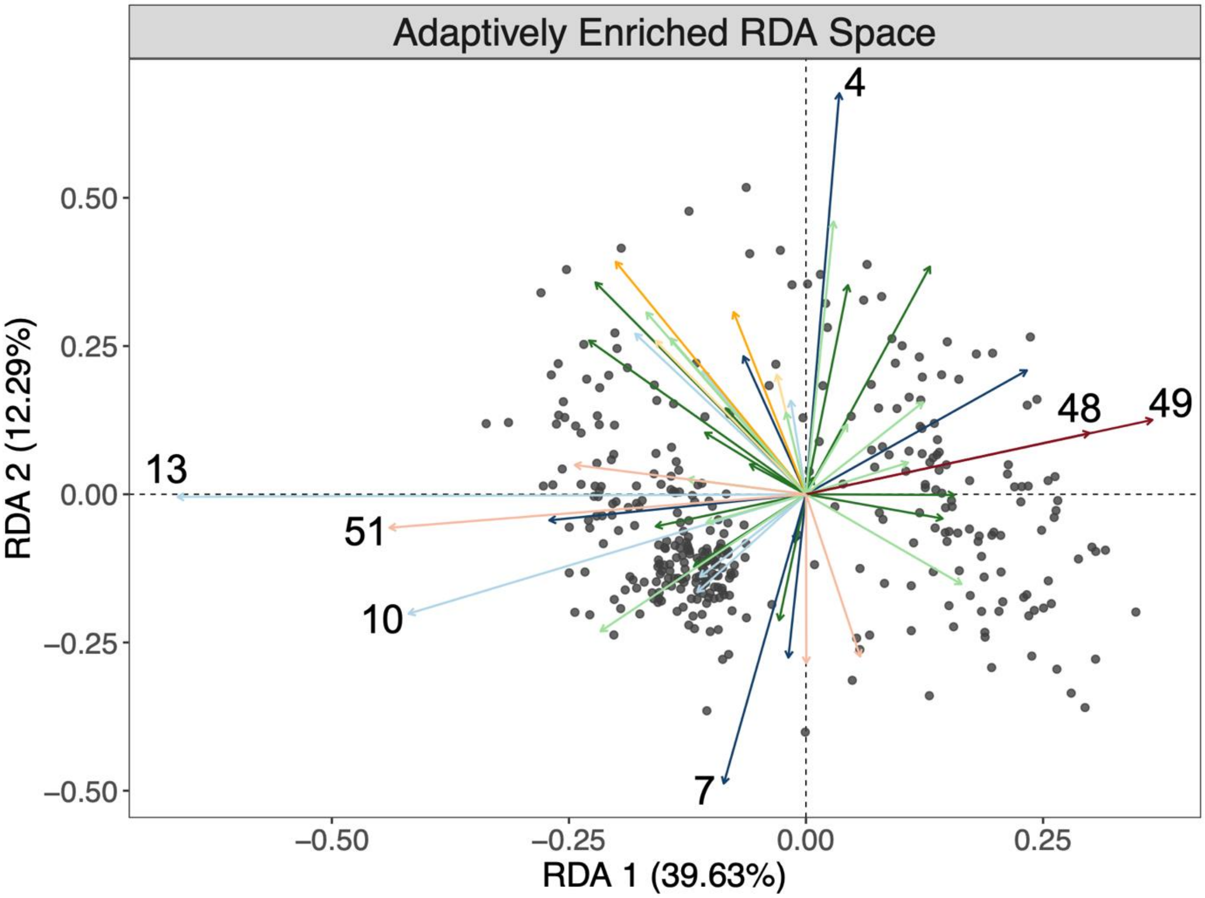

Figure S11. Results from aeRDA conducted using the dataset inclusive of locally derived environmental variables for waterfalls. Vectors are color-coded as in Table 1, and numeric labels match the field numbers listed in Table S2.

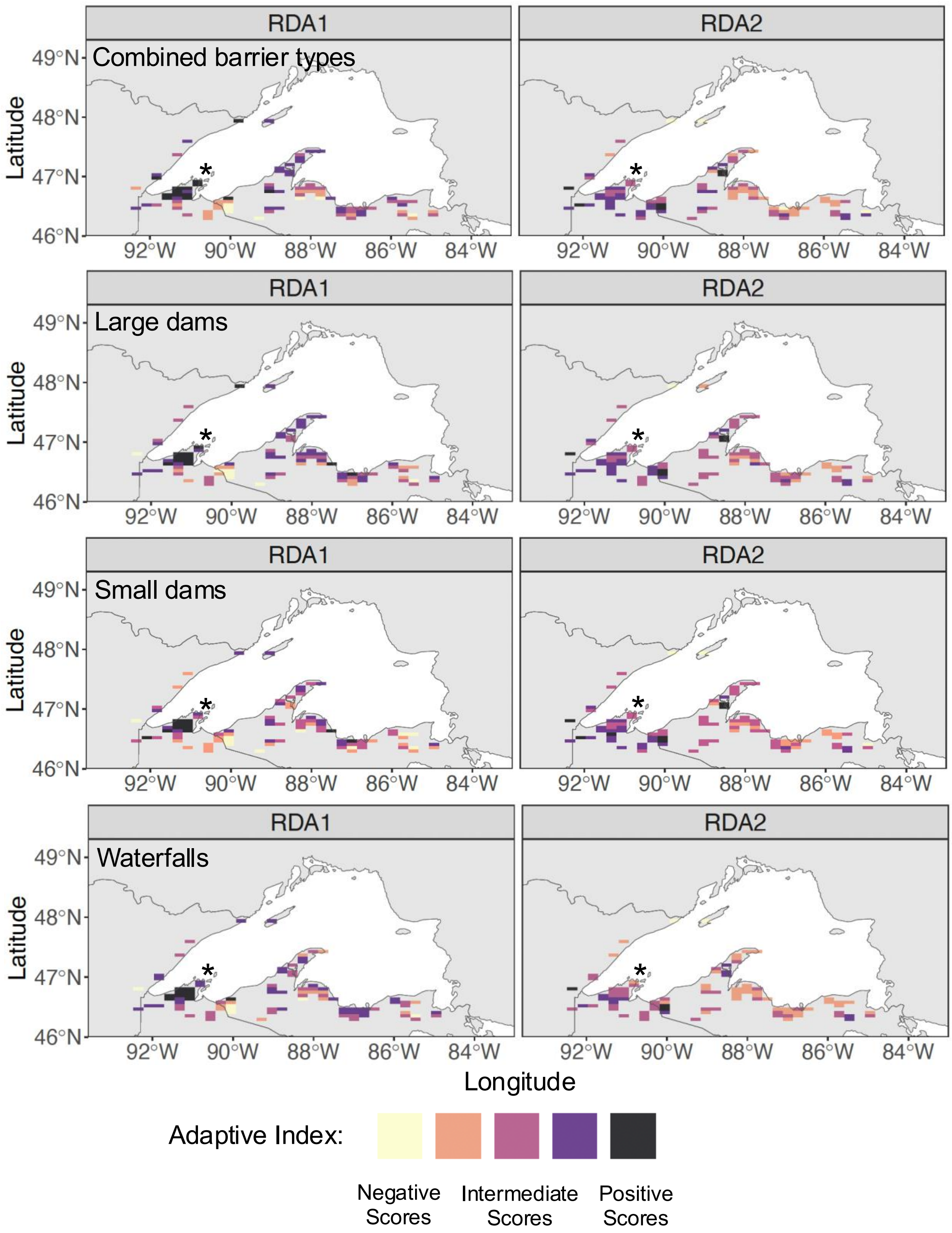

Figure S12. Contemporary landscapes based on adaptive index calculated from aeRDA conducted using the dataset inclusive of locally derived environmental variables. Results are shown for each barrier dataset. Pixels correspond with sampling sites within the Lake Superior basin. The location of the Bayfield Peninsula is indicated with asterisks.
